# Technological Integration in Aesthetic Practice: A Systematic Review of Artificial Intelligence, Augmented Reality and Robotics in Cosmetic Procedures

**DOI:** 10.1007/s00266-026-05898-w

**Published:** 2026-05-15

**Authors:** Raffaele Aguglia, Tobias Niederegger, Yannick Sprunger, Leonard Knoedler, Javier Gonzalez, Curtis L. Cetrulo, Alexandre G. Lellouch, Diala Haykal

**Affiliations:** 1 Division of Plastic and Reconstructive Surgery, Cedars-Sinai Medical Center, Los Angeles, CA USA; 2 University of Heidelberg, Medical Faculty, Heidelberg, Germany; 3 Plastic and Reconstructive Surgery Department, Hôpital Européen Georges Pompidou, Paris, France; 4 Department of Oral and Maxillofacial Surgery, Charité–Universitätsmedizin Berlin, corporate member of Freie Universität Berlin and Humboldt-Universität zu Berlin, Augustenburger Platz 1, 13353 Berlin, Germany; 5 Inserm, The Paris Cardiovascular Research Center, Team Endotheliopathy and Hemostasis Disorders, Université Paris Cité, Paris, France; 6 Innovative Therapies in Haemostasis, INSERM UMR-S 1140, University of Paris, 75006 Paris, France; 7 Centre Laser Palaiseau, Palaiseau, France; 8 Division of Plastic and Reconstructive Surgery, Cedars-Sinai Medical Center, 8700 Beverly Boulevard, Los Angeles, CA 90048 USA

**Keywords:** Cosmetic procedures, Artificial intelligence, Augmented reality, Robotics, Outcome simulation, Cosmetic surgery, Aesthetic medicine

## Abstract

**Background:**

Artificial intelligence (AI), augmented reality (AR), and robotics are rapidly transforming aesthetic practice. Despite their growing integration, evidence on their clinical applications, performance, and challenges in cosmetic procedures remain fragmented. This study systematically reviews clinical applications of these tools.

**Methods:**

A systematic review was conducted following PRISMA 2020 guidelines and registered in PROSPERO (CRD420251077168). Comprehensive searches of PubMed/MEDLINE, EMBASE, Web of Science, and Google Scholar identified original studies on AI, AR, or robotics in aesthetic procedures. Eligible studies included applications in surgical and non-surgical contexts.

**Results:**

From 12,316 screened studies, 55 (0.5%) met the eligibility criteria, published between 2009 and 2025. Most studies (n = 33, 60%) applied AI-based image analysis, enabling objective quantification of skin features, volumetric planning in breast and facial surgery, and improved patient communication. A smaller proportion (n = 5, 9.1%) focused on AI-driven risk assessment and outcome prediction. Robotics (n = 9, 16%) could enhance precision in facial, mandibular, hair, and laser procedures, and outperform manual techniques. AR (n = 8, 15%) allowed intraoperative navigation, and preoperative simulations. Methodological quality was overall low-to-moderate.

**Conclusion:**

While AI is rapidly advancing, offering software capable of comprehensive skin analysis, improving patient selection, predicting outcomes, and aiming to objectively assess the results of aesthetic procedures, AR and robotics have been slower to gain a foothold in cosmetic medicine and surgery. Although some studies highlight the remarkable potential of these technologies, their integration into routine practice remains hindered by limitations in evidence quality, dataset diversity, workflow adaptability, cost, and ethical oversight.

**No Level Assigned:**

This journal requires that authors assign a level of evidence to each submission to which Evidence-Based Medicine rankings are applicable. This excludes Review Articles, Book Reviews, and manuscripts that concern Basic Science, Animal Studies, Cadaver Studies, and Experimental Studies. For a full description of these Evidence-Based Medicine ratings, please refer to the Table of Contents or the online Instructions to Authors www.springer.com/00266.

**Supplementary Information:**

The online version contains supplementary material available at 10.1007/s00266-026-05898-w.

## Introduction

The field of aesthetic medicine and surgery is undergoing a profound transformation, driven by rapid technological advancements that are redefining the way practitioners approach cosmetic care. Over the past decade, artificial intelligence (AI) has emerged as a pivotal force in healthcare, streamlining patient management through enhanced efficiency, safety, and workflow optimization [1–7]. The integration of AI into aesthetic medicine has progressed rapidly over the past two decades. Initially limited to facial recognition and basic imaging tools, its foundation was laid in consumer technology, most notably through beautifying photo filters on social media, which shaped self-perception and raised expectations around cosmetic outcomes [8]. As digital aesthetics became mainstream, AI evolved from image enhancement to clinical utility. Today, AI-driven platforms support facial analysis, procedural simulation, and data-informed decision-making, transforming both the patient experience and practitioner workflows. What began as a consumer trend has matured into a clinical asset, now central to assessment, planning, and outcome evaluation in aesthetic care [1–3, 9, 10].

Historically, within AI, early computer vision methods relied primarily on rule-based image analysis using handcrafted feature extraction and deterministic algorithms, commonly referred to as classical computer vision. While effective for detecting predefined visual patterns, these approaches were limited in their ability to generalize across complex datasets. Central to this evolution is machine learning (ML), a subset of AI that enables computers to recognize complex patterns by processing vast datasets [11]. This foundation has paved the way for more sophisticated software based on deep learning (DL) and artificial neural networks (ANN), including convolutional neural networks (CNN), which are now integral to medical image analysis and diagnostic precision [12]. These innovations have significantly augmented clinical decision-making, particularly in tasks such as differentiating between benign and malignant skin lesions or detecting abnormalities in radiological scans [13, 14].

Robotic-assisted surgery has proven effective across numerous specialties, becoming a routine practice that enhances surgical safety and precision while also helping to reduce associated costs [15]. Concurrently, augmented reality (AR), positioned at the nexus of AI and robotics, has introduced new possibilities in surgery and education by overlaying digital content onto the physical environment, offering surgeons real-time visual enhancements and planning tools [16–18].

This technological shift has coincided with a growing global demand for anti-aging and aesthetic procedures, catalyzing the development of intelligent systems tailored to the unique needs of aesthetic practice [9, 19, 20]. As AI, AR, and robotic platforms become increasingly embedded in the clinical workflow, their integration into aesthetic consultations and interventions has become a subject of both innovation and inquiry. Notably, a recent consensus within the aesthetic medicine community has endorsed the ongoing development and application of these technologies, recognizing their transformative potential in both non-invasive and surgical contexts [21]. However, this progress is not without ethical complexity. The use of intelligent technologies in appearance-related interventions raises important questions around privacy, bias, informed consent, and the evolving doctor–patient relationship [22].

Given the momentum of these developments, this systematic review seeks to examine how digital tools are being integrated into modern aesthetic consultations. It aims to explore their role in enhancing cosmetic procedures before, during, and after intervention, while also addressing the opportunities and challenges they may present for future practice. The novelty of this review lies in its comprehensive synthesis of current evidence, serving as a potential up-to-date reference for aesthetic professionals navigating the evolving landscape of technology-enhanced care.

## Methods

This systematic review adhered to the 2020 Preferred Reporting Items for Systematic Reviews and Meta-Analyses (PRISMA) guidelines. Anticipating considerable variation in study designs and reported outcomes, the authors employed a qualitative synthesis rather than a meta-analysis. The complete review protocol was pre-registered in PROSPERO (ID: CRD420251077168).

### Systematic Search

A comprehensive literature search was conducted in PubMed/MEDLINE, EMBASE, Web of Science, and Google Scholar (first 10 pages) for studies published through August 2025. The search strategy combined two primary concept categories with the Boolean operator “AND”: 1) “artificial intelligence OR robotic surgery OR augmented reality” and 2) “cosmetic procedures.” Relevant synonyms and database-specific terms were applied for each concept. Reference lists of included articles were also reviewed to identify additional eligible studies through cross-referencing. (Supplementary Digital Content 1)

Studies were eligible if they reported original peer-reviewed research (prospective or retrospective) on the use of AI, AR, or robotic technologies in cosmetic procedures. Inclusion criteria encompassed applications in procedural planning, outcome simulation, intraoperative support, skin analysis, patient assessment, or treatment personalization, as well as evaluations of clinical outcomes, workflow efficiency, or safety improvements in cosmetic or reconstructive practice. Exclusion criteria included non-original works (e.g., systematic or narrative reviews, letters, conference abstracts), studies unrelated to cosmetic procedures, those lacking peer-review, duplicate data, or insufficient methodological detail for critical appraisal.

**Fig. 1:**
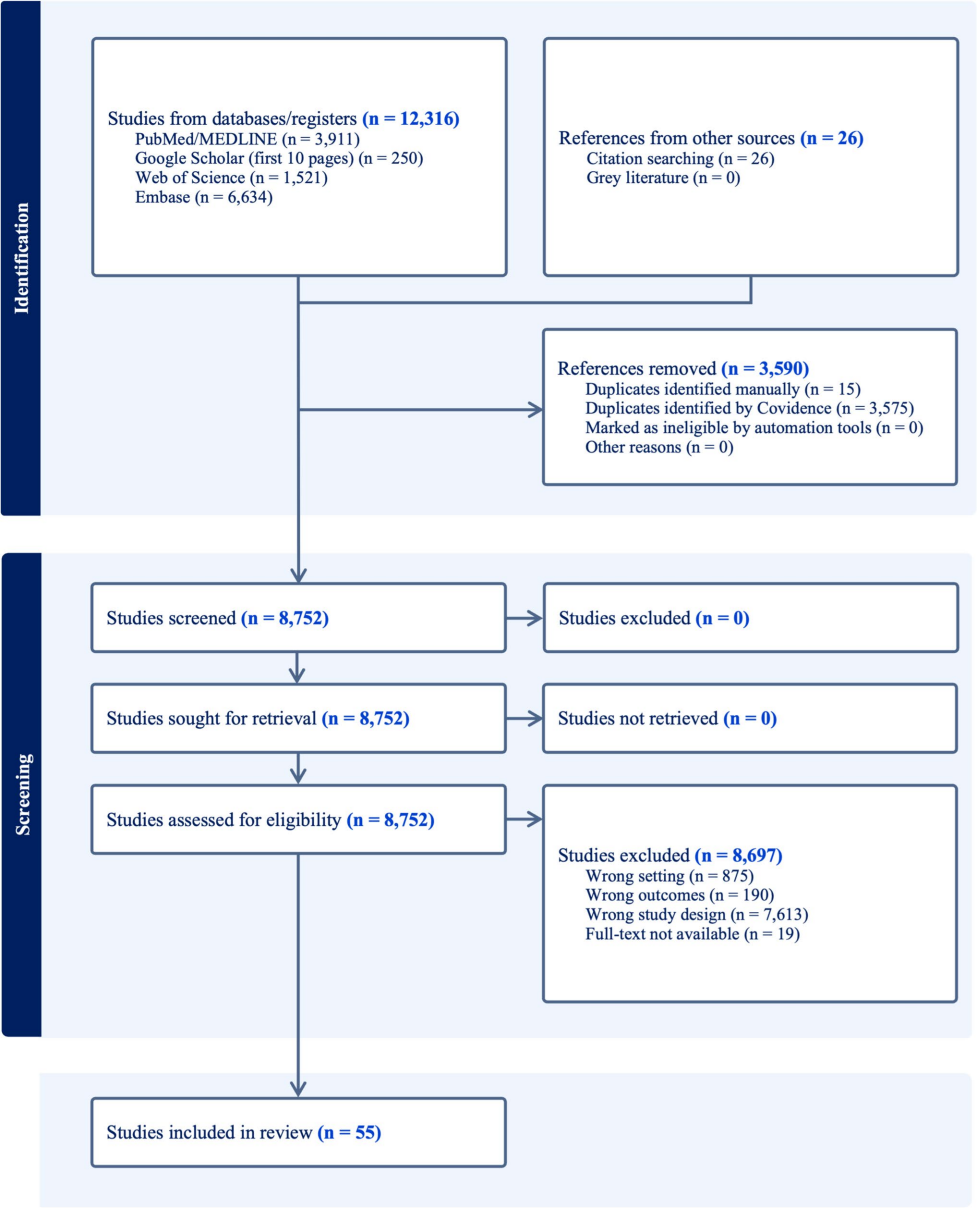
PRISMA 2020 flowchart, highlighting the study selection process

Screening of titles and abstracts was performed independently by three reviewers (R.A., T.N., Y.S.), followed by detailed full-text assessment. Disagreements were resolved in consultation with a senior reviewer (A.G.L., D.H.). The selection process is presented in the PRISMA 2020 flow diagram (Fig. 1).

### Quality Assessment

The certainty of evidence across outcomes was evaluated using the Grading of Recommendations Assessment, Development and Evaluation (GRADE) framework to provide a structured assessment of methodological limitations, inconsistency, indirectness, imprecision, and potential publication bias in the absence of quantitative synthesis [23]. In addition, the methodological quality of each included study was evaluated using the Newcastle–Ottawa Scale (NOS) for clinical research, which assesses three key domains: (i) selection of study cohorts, (ii) comparability between groups, and (iii) determination of outcomes. The NOS awards up to nine stars, with higher scores reflecting superior quality [24]. In addition, the Oxford Centre for Evidence-Based Medicine Levels of Evidence (LOE) framework was applied to standardize evidence grading, designating randomized controlled trials and systematic reviews as Level I evidence, while appraising observational studies and case reports according to their clinical significance and practical utility [25]. Detailed quality assessment results are available in Supplementary Digital Content 2 and 3.

### Data Extraction

To maintain consistency and minimize bias in data collection, a standardized double-review extraction process was used. For each included study, researchers systematically recorded and cross-verified the following parameters: Digital Object Identifier, last name of the first author, study title, study type, year of publication, region of publication, procedure type (e.g., surgical technique, laser application, targeted body area, skin analysis), type of AI, AR or robotics utilized, AI role in surgical mapping, procedure planning, and/or outcome prediction, description of how robotics were integrated into surgical and non-surgical procedures, AI applications in risk prediction of aesthetics, patient safety, AR application, overall study outcomes, and a one-sentence synopsis emphasizing the role of AI, AR or robotics in cosmetic procedures or outcome assessment.

## Results

**Fig. 2:**
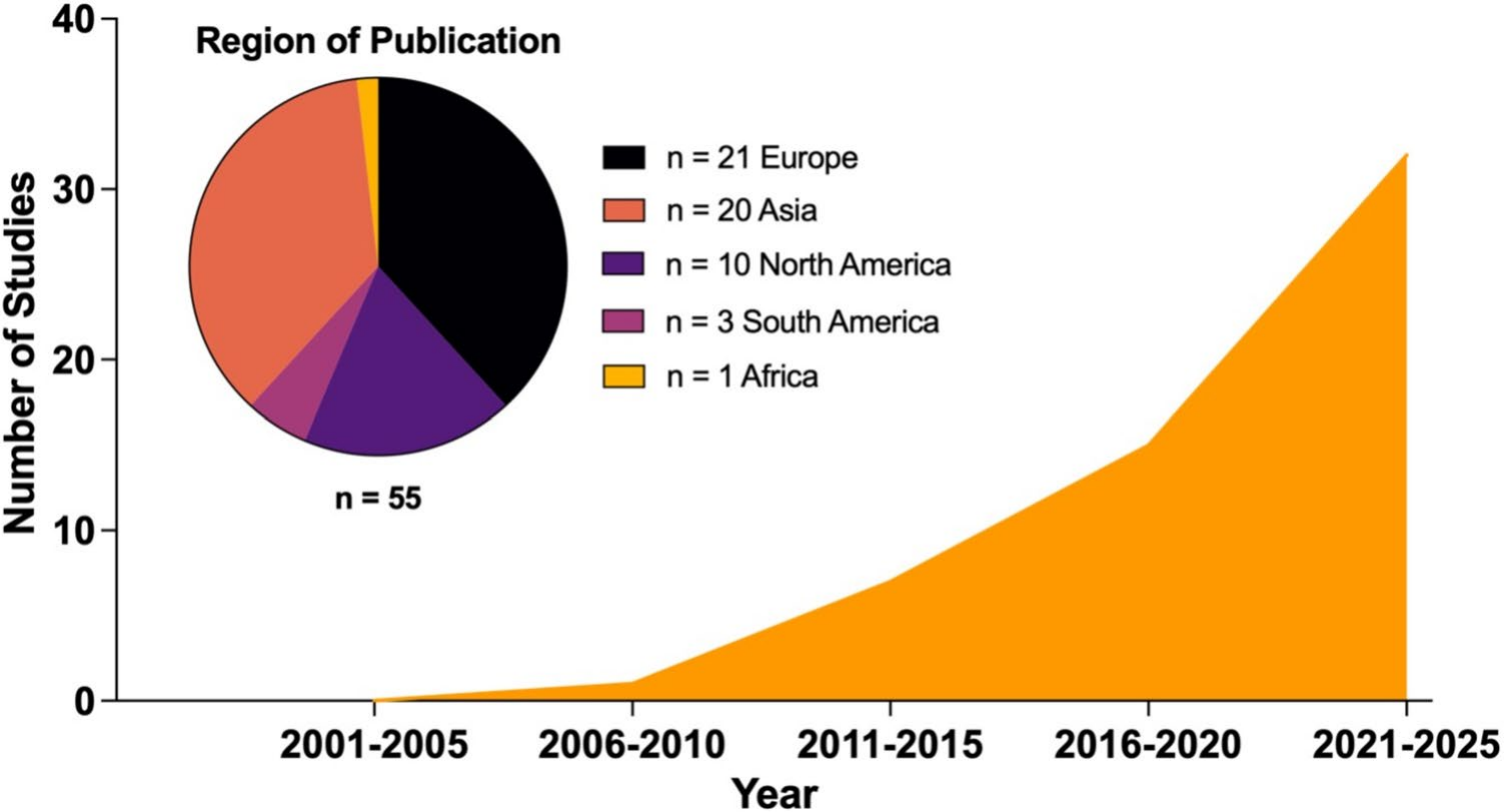
Region of publication distribution and year of publication progression, supporting an increased relevance for AI, augmented reality and robotics in cosmetic procedures

**Fig. 3:**
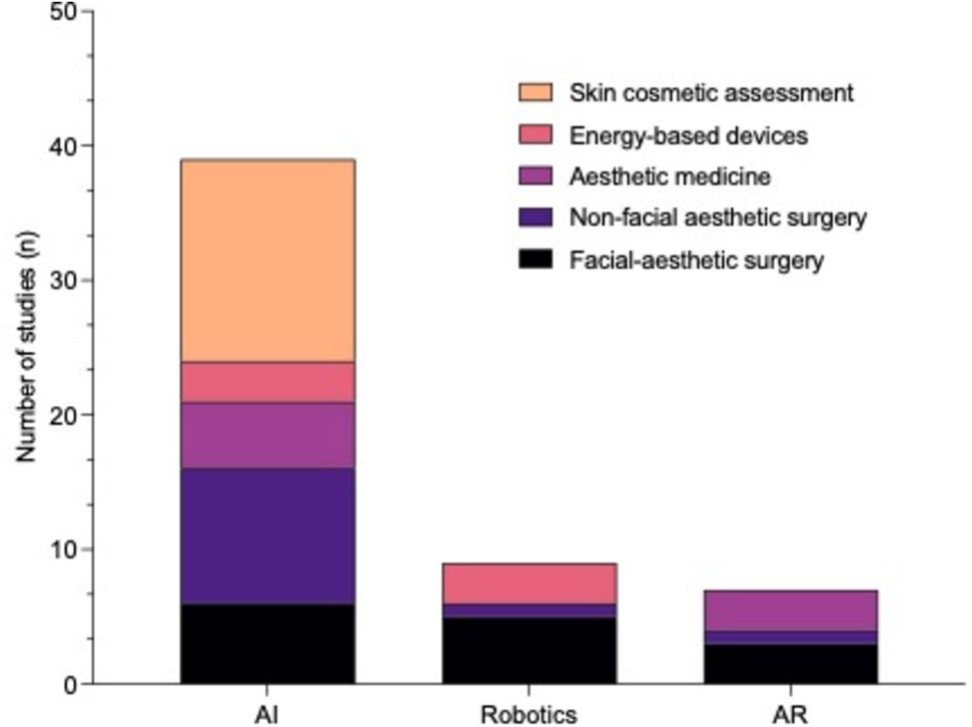
Distribution of AI, robotics, and AR peer-reviewed studies across cosmetic procedure fields

Most studies were conducted in Europe (n = 21, 38%), Asia (n = 20, 36%) and North America (n = 10, 18%) (Fig. 2). Of the included studies, n = 33 (60%) utilized AI based on patient imaging approaches, n = 5 (9.1%) applied AI for individual patient risk assessment, outcome prediction and intraoperative adaption of surgical strategy. Among the included studies, n = 17 (31%) employed classical computer vision techniques, n = 9 (16%) used ML models, n = 6 (11%) implemented DL architectures, and n = 6 (11%) specifically relied on neural network-based approaches such as ANN or CNN. Nine (16%) studies investigated the use of robotics in cosmetic procedures, and n = 8 (15%) examined applications of AR in cosmetic procedures (Fig. 3). The certainty of evidence using the GRADE framework, ranged from low to moderate, primarily due to limitations in study design and limited external validation of several technologies. Detailed outcome-level GRADE assessments and study design classifications are provided in the Supplementary Digital Content 4. The LOE ranged from Level 5 (Foundational Evidence) to Level 1b. The mean (SD) NOS-score was 4.9 (1.5), indicative of overall low-to-moderate methodological rigor. Full demographics are provided in Supplementary Digital Content 5.

### AI-Enhanced Patient Image Analysis

#### Image-Based AI-Enhanced Skin Analysis

Machine learning and DL-based image analysis systems are now central to skin cosmetic assessment. Modern systems combine imaging modalities, from standard 2D photography to high-resolution 3D mapping, with advanced ML models, such as CNNs, generative adversarial networks [26], and transformer architectures. Platforms including VISIA®, OBSERV® 520x, LifeViz® Micro, and ML-enabled apps accurately quantify wrinkles, pigmentation, vascular lesions, pore size, and surface texture [27–31]. Huang et al. found OBSERV® 520x more clinically aligned with MASI pigmentation scores than VISIA®, while Linming et al. showed ANTERA 3D®’s superior wrinkle and pore detection, highlighting inter-device variability [27, 29].

Wrinkle analysis has progressed from Gabor-based morphology to hybrid enhancement algorithms and real-time, expression-sensitive systems like VIS [30, 32, 33]. Beyond diagnosis, convolutional neural networks support treatment monitoring and patient consultation: Alrabiah et al. used Inception-v3 + MTCNN to classify forehead wrinkles for filler planning (85% accuracy), Georgievskaya et al. introduced age and gender-adjusted scoring for personalized benchmarking, and Kang et al. combined landmark detection, age estimation, and emotion recognition to assess lip filler outcomes, showing reduced perceived age and improved facial expression balance [34–36]. In facial surgery, CAARISMA® ARMM provides objective postoperative aesthetic scoring in facial cosmetic procedures [2]. Further information is provided in Table 1.

**Table 1:** Applications of artificial intelligence in cosmetic procedures. This table summarizes studies applying AI in cosmetic contexts

DOI	First author	Title	Year	Type of AI	Role of AI	Overall study outcomes
https://doi.org/10.1016/j.patcog.2014.08.003	Batool et al.	Fast detection of facial wrinkles based on Gabor features using image morphology and geometric constraints	2015	Classical computer vision	Gabor filter-based feature extraction with deterministic image morphology algorithms applied for wrinkle detection	Wrinkle detection 25–85% (typical 60–75%); FAR 0.01–0.33%; faster than MPP (9 s vs. 65 s)
https://doi.org/10.2147/CCID.S135441	Kerscher et al.	Effectiveness evaluation of two volumizing hyaluronic acid dermal fillers in a controlled, randomized, double-blind, split-face clinical study	2017	Classical computer vision	LifeViz® 3D system (QuantifiCare) applied for longitudinal anatomical volume measurements at M3, M6, M12, and M18	Dermal filler CPM-26 superior to VYC-20 (GAIS, volume, longevity up to 18 mo); both safe
https://doi.org/10.1111/srt.12381	Linming et al.	Comparison of two skin imaging analysis instruments: The VISIA® from Canfield vs the ANTERA 3D®CS from Miravex	2018	Classical computer vision	3D-imaging and planning system	ANTERA 3D® more sensitive than VISIA® for wrinkles/pores; both correlated in color analysis
https://doi.org/10.1111/srt.12464	Petit et al.	Validation of 3D skin imaging for objective repeatable quantification of severity of atrophic acne scarring	2018	Classical computer vision	LifeViz® Micro with Mountains Maps® software used for 3D imaging and analysis of facial skin with atrophic acne scarring	Acne scar imaging: strong clinical correlation (r = 0.77), excellent repeatability (ICC = 0.98), robust to acne/lighting
PMID: 29601620	Taub et al.	Multi-Center, Double-Blind, Vehicle-Controlled Clinical Trial of an Alpha and Beta Defensin-Containing Anti-Aging Skin Care Regimen	2018	Classical computer vision	QuantifiCare 3D imaging coupled with Cortex DermaLab Suite used with AI for outcome assessment: skin imaging, elasticity, hydration, and pigmentation analysis	Skincare regimen improved epidermal thickness, wrinkles, pores, pigmentation, evenness
https://doi.org/10.1016/j.jormas.2019.03.012	Savoldelli et al.	Accuracy, repeatability and reproducibility of a handheld three-dimensional facial imaging device: The Vectra H1	2019	Classical computer vision	VECTRA H1 3D-imaging and planning system	Imaging device validated: high accuracy, repeatability, reproducibility
https://doi.org/10.14569/IJACSA.2019.0100941	Alrabiah et al.	Computer-based Approach to Detect Wrinkles and Suggest Facial Fillers	2019	CNN	DL (Inception-v3 + MTCNN) applied for facial landmark detection, classifying images as “Wrinkles” or “No Wrinkles” to guide filler injection decisions	85.3% accuracy for forehead wrinkle detection
https://doi.org/10.3390/jimaging6040017	Elbashir et al.	Evaluation of Automatic Facial Wrinkle Detection Algorithms	2020	Classical computer vision	AI-based wrinkle detection (Hybrid Hessian, Gabor, Enhancement methods) applied for outcome assessment, enabling detection and quantification of facial wrinkles for aging analysis and soft biometrics	Enhancement method best wrinkle detection (JSI 56%) vs. Hessian (32%) and Gabor (16%)
https://doi.org/10.1093/asj/sjz259	Dorfman et al.	Making the Subjective Objective: Machine Learning and Rhinoplasty	2020	DL	Ranking CNN via Microsoft Azure Face API applied for age estimation before and after rhinoplasty to objectively assess facial rejuvenation	Rhinoplasty AI reduced perceived age by 3.1 years
https://doi.org/10.1097/SAP.0000000000002637	Chen et al.	A Comparative Study of Three-Dimensional Simulation in Nonsurgical Rhinoplasty With Hyaluronic Acid Fillers	2021	Classical computer vision	LifeViz® 3D computer simulation used for preoperative nasal angle simulation and quantitative analysis to support planning and outcome comparison	80% of results rated good to excellent; 60% for simulation quality
https://doi.org/10.1093/asj/sjab195	Sforza et al.	Hybrid Implant and Grafted Fat Breast Augmentation: Designing the Pathway to a Future With Breasts Free of Silicone Breast Implants	2021	Classical computer vision	3D imaging and planning system applied for preoperative planning and postoperative volumetric analysis, including fat retention and absorption prediction	Patient satisfaction: 83% excellent, 17% good; Physician satisfaction: 85% excellent, 13% good, 4% fair
https://doi.org/10.1055/s-0041-1729911	Chinski et al.	An Artificial Intelligence Tool for Image Simulation in Rhinoplasty	2021	DL	AI model with GAN architecture used to automatically generate simulated post-rhinoplasty images reflecting surgeon-specific aesthetic criteria for preoperative consultation	68% of participants agreed with AI simulations; 92% correlation with surgeon simulations; AI realistically approximated surgeon’s style
https://doi.org/10.1111/jocd.14106	Holcomb et al.	Helium plasma dermal resurfacing: VISIA CR assessment of facial spots, pores, and wrinkles—Preliminary findings	2021	Classical computer vision	VISIA-CR proprietary image processing algorithms applied for outcome assessment, automating quantification of brown spots, pores, and wrinkles pre- and post-treatment	Brown spots decreased by 45%, pores by 28%, wrinkle area by 13%, and wrinkle thickness by 5%; 96% achieved at least a one-point FWS improvement; 91% reported aesthetic benefit
https://doi.org/10.1016/j.jid.2020.08.027	Dulmage et al.	A Point-of-Care, Real-Time Artificial Intelligence System to Support Clinician Diagnosis of a Wide Range of Skin Diseases	2021	CNN	VisualDx DermExpert AI (ensemble of CNN models incl. DenseNet, NASNetMobile) applied for clinical skin disease diagnosis	AI achieved 68% top-prediction accuracy on a 16-image set and 80% for top three predictions; on a 222-image set, accuracy was 69% overall, consistent across Fitzpatrick types; morphology-specific accuracy ranged from 33% (macule) to 100% (nodule)
https://doi.org/10.2147/CCID.S380388	Zawodny et al.	VISIA Skin Analysis System as a Tool to Evaluate the Reduction of Pigmented Skin and Vascular Lesions Using the 532 Nm Laser	2022	Classical computer vision	VISIA 3D-imaging and planning system	Repetitive 532 nanometer laser therapy significantly reduced vascular and pigmented lesions, with VISIA Skin Analysis confirming improvements across all lesion types
https://doi.org/10.1093/asjof/ojab052	Chartier et al.	BreastGAN: Artificial Intelligence-Enabled Breast Augmentation Simulation	2022	DL	BreastGAN (GAN-based model) applied to simulate postoperative breast augmentation outcomes from preoperative images using image-to-image translation	AI-generated images closely resembled real surgical results, no quantitative assessments were performed
https://doi.org/10.1097/PRS.0000000000009671	Georgievskaya et al.	Artificial Intelligence Confirming Treatment Success: The Role of Gender- and Age-Specific Scales in Performance Evaluation	2022	ANN	AI with computer vision and neural networks used for outcome assessment by analyzing pre- and post-treatment skin conditions and confirming results with age- and gender-specific scoring scales	Analysis of 433 high-quality selfies showed significant gender- and age-related differences in skin parameters, with wrinkles most strongly correlated with age (r = –0.56)
https://doi.org/10.1080/2000656X.2021.2024553	Bai et al.	Clinical assessment of breast symmetry and aesthetic outcome: can 3D imaging be the gold standard?	2023	Classical computer vision	VECTRA XT 3D surface imaging used for breast symmetry and volume assessment following bilateral risk-reducing mastectomy with immediate reconstruction	3D imaging showed substantial to excellent intra-observer reproducibility for symmetry and volume, moderate to substantial inter-observer agreement, and high inter-posture consistency; the proposed VSS metric demonstrated good reliability, no strong correlation was found between breast symmetry and volume differences
https://doi.org/10.1038/s41598-023-49006-3	Knoedler et al.	Diagnosing lagophthalmos using artificial intelligence	2023	CNN	Custom CNN (AlexNet-based) used for diagnostic support by identifying lagophthalmos from standardized facial images	AI model demonstrated high accuracy and strong validation and testing performance, with excellent precision, specificity, and overall reliability, while training was completed efficiently within a short time frame
https://doi.org/10.1111/srt.13206	Fujino et al.	Real-time wrinkle evaluation method using Visual Illusion-based image feature enhancement System	2023	Classical computer vision	Unsupervised VIS system adapted for wrinkle detection, enhancement, and quantitative measurement from static and dynamic facial images	VIS detected fine wrinkles invisible to the naked eye, confirmed age-related wrinkle increase, and showed slight but non-significant wrinkle reduction with antiwrinkle cream, while conventional methods confirmed significant improvement under neutral conditions
https://doi.org/10.1186/s13005-024-00406-4	Ritschl et al.	Comparison of three-dimensional imaging of the nose using three different 3D-photography systems: an observational study	2024	Classical computer vision	3D-imaging and planning system	All three imaging systems showed clinically acceptable accuracy, with Artec Space Spider being the most precise and Bellus3D the most practical for routine use
https://doi.org/10.21037/gs-24-148	Lee et al.	Prediction model for intraoperative implant volume using the 3D surface imaging system (VECTRA XT 3D) in direct-to-implant breast reconstructions	2024	ML	VECTRA XT 3D-imaging and planning system	The predictive model improved implant volume selection, leading to good postoperative symmetry and enhanced aesthetic outcomes
https://doi.org/10.1093/asj/sjae040	Demir et al.	Optimizing Implant Width Selection in Breast Augmentation: Insights From On-Patient Landmark Positioning in 3-Dimensional Breast Simulation	2024	Classical computer vision	3D-imaging and planning system	Manual landmark positioning with 3D simulation improved implant width accuracy and planning, though clinical judgment remained most consistent
https://doi.org/10.1038/s41598-024-63274-7	Huang et al.	A comparative study of an advanced skin imaging system in diagnosing facial pigmentary and inflammatory conditions	2024	ML	OBSERV® 520x ML-based imaging system	OBSERV® 520x AI showed moderate to strong correlation with other imaging systems in pigmentation and erythema analysis, significantly correlated with MASI scores, and offered more precise full-face and regional assessments than existing tools
https://doi.org/10.1111/jocd.16218	Lee et al.	Deep learning-based skin care product recommendation: A focus on cosmetic ingredient analysis and facial skin conditions	2024	DL	LUMINI V2 DL with Transformer encoder (ingredient analysis) and U-Net segmentation (skin analysis) applied for AI-based skincare product recommendation	LUMINI V2 skin analyzer showed strong correlation with dermatologist ratings across multiple skin parameters, while the ingredient analyzer achieved high training accuracy but lower performance on validation and test sets
https://doi.org/10.1007/s00266-025-04876-y	Li et al.	An Artificial Intelligence Tool Used for Patient Selection in Cosmetic Surgery	2025	ML	ML with XGBoost algorithm used for preoperative assessment, predicting patient suitability and identifying at-risk individuals before surgery	AI showed high agreement with surgeons on patient suitability and strong correlation with their scoring
https://doi.org/10.1016/j.bjps.2025.02.014	Wang et al.	Can AI-assisted objective facial attractiveness scoring systems replace manual aesthetic evaluations? A comparative analysis of human and machine ratings	2025	DL	DL-based facial analysis with Face++ (CNN-based) applied for objective outcome assessment	AI scores showed good correlation with manual ratings, moderate consistency with human evaluators, and reliable accuracy in classifying facial aesthetics
https://doi.org/10.1007/s00266-025-04891-z	Kang et al.	AI-Driven Assessment of Lip Volume Improvement Using Hyaluronic Acid Fillers: A Comprehensive Analysis	2025	ML	AI tools (LibreFace for landmark detection, MiVOLO for age estimation, FaceReader for emotion recognition) used for post-procedure outcome assessment, including lip volume, age reduction, and emotional expression analysis	AI tools demonstrated high accuracy in age estimation, detected a modest reduction in perceived age, showed shifts toward more neutral and less angry expressions, and visualized improved lip contour and symmetry after treatment
https://doi.org/10.1016/j.jormas.2024.102211	Knoedler et al.	Objective and automated facial palsy grading and outcome assessment after facial palsy reanimation surgery – A prospective observational study	2025	ML	ML-based CAARISMA® ARMM system used for outcome assessment by quantifying postoperative facial aesthetics with indices such as FYI, FAI, and SQI	Facial Aesthetic Index significantly improved, while youthfulness and skin quality showed no overall change; females improved in youthfulness, and younger patients showed better skin quality than older ones
https://doi.org/10.1016/j.jormas.2025.102277	Knoedler et al.	Objectifying aesthetic outcomes following face transplantation – the AI research metrics model (CAARISMA® ARMM)	2025	ML	ML-based CAARISMA® ARMM system used for outcome assessment by quantifying postoperative facial aesthetics with indices such as FYI, FAI, and SQI	Postoperative scores for youthfulness, aesthetics, and skin quality improved significantly, with outcomes consistent across age and gender; no difference was found compared to pre-trauma scores

*DOI* Digital Object Identifier, *IGVD* image-guided visualization display, *AP/ML* anteroposterior and mediolateral, *OR* operating room, *AE* adverse event, *FAR* false alarm rate, *MPP* multivariate polynomial projection, *M3/M6/M12/M18* three/six/twelve/eighteen months, *CPM-26* cohesive polydensified matrix hyaluronic acid filler (26 mg/ml), *VYC-20* Vycross technology hyaluronic acid filler (20 mg/ml), *GAIS* Global Aesthetic Improvement Scale, *mo.* months, *ICC* intraclass correlation coefficient, *r* Pearson’s correlation coefficient, Inception-v3 deep learning CNN architecture, *MTCNN* multi-task cascaded convolutional networks, *JSI* Jaccard similarity index, *VR* virtual reality, *GAN* generative adversarial network, *FWS* Fitzpatrick Wrinkle Scale, *DenseNet* convolutional neural network architecture, *NASNetMobile* convolutional neural network architecture, *nm* nanometer, *VSS* volume-shape-symmetry index, *AUROC* area under the receiver operating characteristic curve, *MASI* melasma area and severity index, *FYI* Facial Youthfulness Index, *FAI* Facial Aesthetic Index, *SQI* Skin Quality Index

#### Volumetric Image-Based AI for Surgical Planning and Guidance in Cosmetic Procedures

Beyond skin surface analysis, 3D analyzers also enable volumetric assessments that help guide aesthetic surgeons. In facial aesthetic surgery, Zinser et al. implemented an image-guided visualization display for waferless orthognathic surgery, enabling millimeter-level accuracy without occlusal splints [37]. In breast surgery, Sforza et al. used computer vision-integrated 3D imaging to plan hybrid implant–fat graft augmentations, while Lee et al. applied VECTRA XT 3D modeling to predict intraoperative implant volume for optimal symmetry [38]. AI-based simulation has also improved pre operative patient communication, as shown by Chinski et al. in rhinoplasty using GAN-generated visual outcomes and by Chartier et al. in breast augmentation with BreastGAN [39, 40]. Deep learning also supports risk prediction and patient selection, with Li et al. employing XGBoost algorithms to preoperatively screen cosmetic surgery candidates [2, 37, 41, 42]. In body contouring, Adjadj et al. used USER ASSIST®, an ML system integrated with the CryoSlim cryolipolysis device, to dynamically adjust treatment parameters based on patient-specific fat characteristics, resulting in significant reductions in thigh circumference and subcutaneous fat, high satisfaction, and minimal adverse effects [43]. Similarly, the Crisalix 3D cloud-based simulation software has been applied in breast surgery, where Vorstenbosch et al. demonstrated moderately accurate predictions of post-augmentation aesthetic outcomes, particularly in symmetric breasts, while Markovic et al. highlighted its value as a low-cost, easy-to-perform, and therefore practical alternative for breast volume estimation [44, 45]. Collectively, these technologies are steering cosmetic practices toward a data-driven paradigm, guiding treatment selection through objective baseline analysis, refining procedural planning with precise image-based metrics, enabling risk stratification, and tracking longitudinal outcomes with quantifiable indices. Detailed insights are shown in Table 1. However, most AI algorithms remain confined to experimental or research settings and have not yet achieved widespread integration into routine clinical workflows. Nevertheless, several platforms have already reached commercial deployment and are used in clinical environments for consultation, treatment planning, or postoperative outcome assessment. A list of commercially available AI-enabled systems reported in peer-reviewed studies is provided in Table 2.

**Table 2:** Comparison of commercial AI-based platforms and applications in cosmetic procedures evaluated in peer-reviewed studies

AI system / platform	Primary use case	Workflow integration	Key applications
ANTERA 3D®	Computer vision-assisted 3D skin imaging with high sensitivity for microtopography	In-office device	Wrinkle and pore detection, fine skin texture analysis
ARBREA®	DL-based AR 3D simulation	Patient consultation and planning	Real-time implant simulation and patient satisfaction prediction
CAARISMA® ARMM	ML-powered outcome scoring	Postoperative workflow	Objective, standardized aesthetic outcome evaluation in facial surgery
CRISALIX®	DL-powered 3D planning and outcome simulation + Pre-surgery VR/AR	Patient consultation	Pre-surgical visualization, predictive analytics
LifeViz® Micro	Computer vision-assisted 3D high-resolution skin mapping	Clinic-based handheld 3D system	Scar severity assessment, acne scar quantification, skin texture evaluation
OBSERV® 520x	ML-multi-modal skin imaging (surface and subsurface)	Non-invasive imaging system	Pigmentation (MASI correlation), vascular lesions, treatment monitoring
USER ASSIST® (CryoSlim)	ML-feedback integrated into cryolipolysis device	Intraoperative (body contouring)	Fat parameter analysis (volume, density, consistency) with real-time treatment adjustment
VECTRA® XT 3D	AI-driven 3D surface imaging and surgical planning when implemented with VECTRA analysis module	Preoperative workflow and intraoperative guidance	Implant volume prediction, symmetry analysis, surgical planning
VISIA®	2D skin imaging with ML wrinkle, pore, and pigmentation quantification	Outpatient consultation and follow-up	Skin quality analysis, wrinkle/pore monitoring, treatment tracking

### Advancements and Clinical Applications of Robotics in Cosmetic Procedures

Facial cosmetic surgery is a good example of the potential applications of robotics. Promising cadaveric studies demonstrated precise, tremor-free platysmaplasty through facelift incisions while Smith et al. achieved sub-millimeter precision (RMSE ≤ 0.43 mm) in rib cartilage sculpting for nasal tip reconstruction, shortening carving time by up to 8 min [46, 47]. In mandibular contouring, Lin et al. employed the CPSR-I system with AR-driven force feedback, reporting mean drilling errors of 1.38 mm, no complications, and high patient satisfaction [48]. Minimally invasive cosmetic treatments have similarly advanced. Robotic laser hair removal systems by Lim et al. and Park et al. delivered uniform, sub-millimeter accuracy and achieved greater hair reduction than physician-led treatments (49% vs. 30%) [49–51]. In hair restoration, Zhu et al. found the ARTAS AI-guided system matched manual FUE yield rates (82% vs. 90%) with reduced transection for specific follicle types but higher discard rates thanks to ML-supported follicle identification [52]. Importantly, most robotic applications identified in aesthetic procedures remain at a preclinical or early pilot stage, with only a limited number of commercially deployed systems, such as robotic hair restoration platforms. Collectively, these studies highlight how a broader integration of robotics in cosmetic procedures could achieve high technical precision, smaller incisions or access points, shorter recovery times, and superior aesthetic satisfaction. The full data is provided in Table 3.

**Table 3:** Summary of robotics applications in cosmetic procedures

DOI	First author	Title	Year of publication	Type of robotics	Application of robotics	Overall study outcomes
https://doi.org/10.1007/s11701-013-0431-2	Taghizadeh et al.	Evaluation of robotic-assisted platysmaplasty procedures in a cadaveric model using the da Vinci Surgical System	2014	da Vinci Si Surgical System	Deep platysma dissection and suturing in platysmaplasty	Robotic-assisted platysmaplasty was feasible, reproducible, and improved lower neck lift outcomes using minimally invasive techniques
https://doi.org/10.1089/pho.2014.3774	Lim et al.	A Study on the Development of a Robot-Assisted Automatic Laser Hair Removal System	2014	Six-axis industrial robot: Equipped with webcam and laser sensor for vision-guided tasks	Vision-based detection and mapping enabled uniform laser hair removal via real-time camera input and laser-guided robotic motions	Robotic system achieved ~1 mm localization error, uniform laser delivery (~38 mm^2^/spot), rapid treatment (< 40 s), and high consistency
https://doi.org/10.1245/s10434-013-3470-z	Mohamed et al.	Retroauricular Robotic Thyroidectomy with Concomitant Neck-Lift Surgery	2015	Robotic-assisted surgery: General robotic platform for surgical procedures	Robotic thyroidectomy via retroauricular incision; concomitant neck-lift performed manually through same access	Robotic thyroidectomy via retroauricular incision was safe, feasible, and improved cosmetic outcomes with concurrent neck-lift
https://doi.org/10.1089/pho.2015.3948	Lim et al.	Comparison of Efficacy Between Novel Robot-Assisted Laser Hair Removal and Physician-Directed Hair Removal	2015	Automatic laser hair removal robot: Robotic system for laser-based depilation	Robotics automated laser delivery, ensuring uniform treatment distribution to target skin area	Robot-assisted LHR achieved higher hair removal but required longer treatment and more shots; no adverse effects
https://doi.org/10.1089/pho.2015.4018	Park et al.	Improvement in Laser-Irradiation Efficiency of Robot-Assisted Laser Hair Removal Through Pose Measurement of Skin Surface	2016	Laser hair removal robot with pose algorithm: Hair removal robot enhanced with pose-measurement for accuracy	Algorithm calculated 6-DOF skin pose for optimized laser contact	Robotic system maintained accurate contact along curved skin, demonstrating precise LHR delivery
https://doi.org/10.1038/s41598-021-85889-w	Lin et al.	Preliminary clinical experience of robot-assisted surgery in treatment with genioplasty	2021	CPSR-I: 7-DOF craniofacial surgical robot with fuzzy control and force feedback	Automated osteotomy drilling	Robotic osteotomy showed low drilling errors, safe outcomes with no complications, and high patient satisfaction
https://doi.org/10.1097/PRS.0000000000008670	van Mulken et al.	One-Year Outcomes of the First Human Trial on Robot-Assisted Lymphaticovenous Anastomosis for Breast Cancer-Related Lymphedema	2022	MUSA (MicroSure, Netherlands): Microsurgical robot with master–slave control, motion scaling, and tremor filtration for high-precision tasks	Robotics used only for anastomosis	Robot-assisted supermicrosurgery was feasible with comparable outcomes to manual surgery in quality of life and patency
https://doi.org/10.1097/SCS.0000000000009272	Smith et al.	Robot-Assisted Nasal Reconstruction: A Cadaveric Study	2023	Augmented robotic burring system: Robot with spherical burr for bone procedures	Robotics used for scanning, 3D modeling, and automated rib cartilage carving for nasal reconstruction, guided by preoperative 3D models	Robotic cartilage carving achieved high precision, faster than manual carving, with consistent efficiency
https://doi.org/10.1111/jocd.16554	Zhu et al.	A Comparative Study on the Application of Robotic Hair Restoration Technology Versus Traditional Follicular Unit Excision in Male Androgenetic Alopecia	2024	ARTAS: Robotic hair transplant system with AI imaging and follicle recognition	ARTAS robotic system was used for autonomous follicular unit recognition and extraction, assessing direction, angle, and distribution without human intervention	ARTAS hair transplantation had similar outcomes to FUE, with higher discard rates but lower transection rates for certain follicular units

*DOI* Digital Object Identifier, *LHR* laser hair removal, *6-DOF* Six Degrees of Freedom, *7-DOF* Seven Degrees of Freedom, *CPSR-I* Craniofacial-Plastic Surgical Robot I, *AR* augmented reality, *MUSA* Microsure Surgical System, *AI* artificial intelligence, *FUE* follicular unit excision

### Augmented Reality in Cosmetic Procedures

AR technologies have emerged as valuable tools in cosmetic and maxillofacial surgery, offering enhanced visualization, surgical precision, and patient-specific planning without disrupting procedural workflow [53–56]. In orthognathic applications, Badiali et al. validated the WARM head-mounted AR device for waferless LeFort I maxillary repositioning, achieving mean positional errors under 2 mm across varying plan complexities, independent of operator experience [57]. Similarly, Wang et al. developed a GPU-accelerated, marker-less AR navigation system, attaining ~1 mm registration accuracy for oral and maxillofacial surgical navigation [56]. AR has also been combined with robotics, as shown by Lin et al., where robot-assisted arms executed AR-planned drilling paths for mandibular angle split osteotomy with significantly reduced positional and angular errors compared to manual methods [55].

In facial plastic and non-surgical cosmetic procedures, AR facilitates both intraoperative assessment and complication prevention. In injection-based treatments, Kim et al. applied DL-driven AR facial mapping to guide botulinum toxin placement with sub-millimeter accuracy, while Mespreuve et al. and Waked et al. used MRA-based AR visualization to project patient-specific facial arterial anatomy, aiming to minimize risks such as necrosis and blindness during dermal filler injections [53, 58, 59]. Beyond safety, AR also enhances patient engagement and satisfaction, as evidenced by La Padula et al., where AI-based AR simulations in breast augmentation improved preoperative planning and significantly increased BREAST-Q scores [54]. The use of virtual reality has been reported for training in filler injections, improving both safety and awareness of anatomical landscapes in medical trainees [60]. Collectively, these studies underscore AR’s growing role in cosmetic medicine as a precision-enhancing, safety-oriented, and patient-centered technology. However, most AR systems reported in the literature remain pilot or research-stage technologies, with limited routine clinical deployment outside specialized research settings. Further insights are shown in Table 4.

**Table 4:** Summary of augmented reality (AR) applications in cosmetic procedures

DOI	First author	Title	Year of publication	Type of augmented reality	Application of augmented reality	Overall study outcomes
https://doi.org/10.1016/j.bjoms.2013.06.014	Zinser et al.	Computer-assisted orthognathic surgery: waferless maxillary positioning, versatility, and accuracy of an image-guided visualization display	2013	AR navigation for preoperative virtual surgical planning	Image-guided visualization displays (IGVD, e.g., I-plan CMF®, BrainLab; Vector Vision2) enable virtual surgical planning, intraoperative navigation, real-time maxilla repositioning, and 3D cephalometric outcome analysis	Surgical planning precision: < 0.35 mm (AP/ML),< 0.64 mm vertical; OR time + 60 min; safe, no major AEs
https://doi.org/10.1016/j.jcms.2014.09.001	Badiali et al.	Augmented reality as an aid in maxillofacial surgery: Validation of a wearable system allowing maxillary repositioning	2014	AR wearable system (WARM device) with machine vision alignment	Real-time AR overlay for maxillary repositioning, improving accuracy and ergonomics without external tracking or invasive guides	AR system provided high accuracy across different surgical plan complexities, consistent performance regardless of surgeon experience, and was rated comfortable and easy to use
https://doi.org/10.1002/rcs.1754	Wang et al.	Video see-through augmented reality for oral and maxillofacial surgery	2016	AR with 3D–2D shape matching and TLD algorithm; GPU-accelerated	AI-based AR with 3D–2D image registration for intraoperative guidance, overlaying surgical plans and hidden structures to improve accuracy and safety	AR system demonstrated precise and stable registration with smooth real-time tracking, maintaining accuracy across multiple landmarks in both phantom and clinical testing, while integrating seamlessly into surgical workflow
https://doi.org/10.1016/j.jcms.2015.10.024	Lin et al.	Mandibular angle split osteotomy based on a novel augmented reality navigation using specialized robot-assisted arms – A feasibility study	2016	AR navigation with robot-assisted arms	AR navigation with robotic arms for drilling path planning and real-time anatomical visualization, improving precision and reducing nerve injury risk	Robot-assisted group achieved higher accuracy and stability than manual control, with both groups completing treatment successfully
https://doi.org/10.1002/cpe.5526	Kim et al.	Augmented reality for botulinum toxin injection	2020	AR with deep learning (DL)–based face recognition	AR mapped facial landmarks and guided injection planning to improve accuracy and safety	The AR app demonstrated high recognition accuracy for facial landmarks, supporting its potential use in guiding Botox injections
https://doi.org/10.1007/s00266-020-01872-2	Oh et al.	Development and Usability of a Virtual Reality-Based Filler Injection Training System	2020	Virtual reality (VR)	VR-based system used for visualization and training in filler injections	VR filler training: 76% usability, improved safety/awareness
https://doi.org/10.1093/asjof/ojab018	Mespreuve et al.	The Usefulness of Magnetic Resonance Angiography to Analyze the Variable Arterial Facial Anatomy in an Effort to Reduce Filler-Associated Blindness: Anatomical Study and Visualization Through an Augmented Reality Application	2021	AR navigation	AR projected vascular anatomy to guide filler injections, enhancing safety and preventing complications	MRA combined with AR successfully visualized facial arteries, enhancing safety by guiding filler injections and reducing risk of vascular complications
https://doi.org/10.3390/jcm11123464	La Padula et al.	Assessment of Patient Satisfaction Using a New Augmented Reality Simulation Software for Breast Augmentation: A Prospective Study	2022	Arbrea Breast Software: DL-based 3D AR simulation	AR with DL-based 3D simulation for implant planning and postoperative appearance prediction	Patients reported high satisfaction with AR breast simulations, with significant improvements in satisfaction, psychological well-being, and sexual well-being after surgery
https://doi.org/10.1093/asjof/ojac012	Waked et al.	Visualizing the Individual Arterial Anatomy of the Face Through Augmented Reality—A Useful and Accurate Tool During Dermal Filler Injections	2022	AR navigation	AR with MRA-based facial artery mapping for injection planning; enhanced safety by reducing risk of intravascular complications (necrosis, blindness)	AR projection of facial arteries showed high accuracy compared with ultrasound, visualized numerous vessels safely, and improved guidance for safer filler injections without complications

*DOI* Digital Object Identifier, *AR* augmented reality, *WARM* wearable augmented reality monitor, *3D* three-dimensional, *2D* two-dimensional, *TLD* tracking-learning-detection, *GPU* graphics processing unit, *AI* artificial intelligence, *MRA* magnetic resonance angiography

### AI-Driven Risk Prediction and Outcome Forecasting in Cosmetic Surgery

Machine learning models have increasingly been applied to individualized risk assessment and outcome prediction in cosmetic procedures, improving patient selection and safety. Cazzaniga et al. developed ANN models to forecast treatment response and re-pigmentation time in vitiligo patients undergoing excimer laser therapy [61]. Using lesion-level and demographic data, their discriminant network was more accurate in classifying responders versus non-responders, than classic regression models. In a broader surgical context, Bukret et al. applied a support vector machine (SVM) model across multiple cosmetic procedures (such as breast augmentation, liposuction, and abdominoplasty) to stratify complication risk based on BMI, age, and Caprini-score. This approach enabled surgical risk stratification, contributing to safer patient selection [62]. Montemurro et al. employed a ML-based classification and regression tree (CART) algorithm to identify both established and previously overlooked predictors of complications in primary breast augmentation. Variables such as larger preoperative breast size, taller stature, BMI, and age emerged as significant risk factors, with the model achieving up to 99.8% accuracy in training and 99.1% in testing [63]. Expanding on this work, Huyghebaert et al. used CART, Random Forest, and XGBoost algorithms to evaluate risks in single-stage augmentation mastopexy, identifying BMI, age, smoking status, and implant characteristics as key determinants of complications and implant exchange decisions [64]. Collectively, these studies illustrate how ML-powered models can deliver precise, individualized risk assessments and outcome predictions in cosmetic surgery, enhancing both patient safety and surgical decision-making. Full data is illustrated in Table 5.

**Table 5:** Summary of key studies on AI-driven personalized risk assessment and outcome prediction in cosmetic procedures

DOI	First author	Title	Year of publication	Type of AI	Application of AI	Overall study outcomes
https://doi.org/10.1159/000225934	Cazzaniga et al.	Prediction of Clinical Response to Excimer Laser Treatment in Vitiligo by Using Neural Network Models	2009	ANN	Vitiligo: Predictive modeling of treatment response and repigmentation time using lesion-level and demographic data	Neural network classified vitiligo responders vs. non-responders and predicted repigmentation time with moderate accuracy
https://doi.org/10.1097/PRS.0000000000003433	Adjadj et al.	Assessment of the Efficacy of Cryolipolysis on Saddlebags: A Prospective Study of 53 Patients	2017	ML: CryoSlim USER ASSIST®:	AI-driven system analyzing fat parameters in real time to auto-adjust cryolipolysis settings per patient morphology	CryoSlim reduced thigh circumference and fat thickness with high patient satisfaction, minimal pain, and mild, transient adverse events
https://doi.org/10.1007/s00266-017-0830-2	Vorstenbosch et al.	Correlation of Prediction and Actual Outcome of Three-Dimensional Simulation in Breast Augmentation Using a Cloud-Based Program	2017	Computer vision: Crisalix®: Cloud-based 3D simulation software	Crisalix was used to generate 3D simulations from 2D pre operative images to predict post operative aesthetic results, including breast width, height, projection, volume, nipple correction, and overall appearance	Crisalix-generated simulations were moderately similar to actual outcomes. Simulations were less representative for tuberous and ptotic breasts
https://doi.org/10.1097/GOX.0000000000003698	Bukret et al.	A Novel Artificial Intelligence-assisted Risk Assessment Model for Preventing Complications in Esthetic Surgery	2021	DL: SVM model: Narrow AI using support vector machine for classification tasks	AI model stratified complication risk using patient factors (BMI, age, Caprini score)	AI predicted surgical complications by stratifying risks (age, BMI, Caprini score), supporting safer patient selection
https://doi.org/10.1007/s00266-022-02997-2	Montemurro et al.	A Machine Learning Approach to Identify Previously Unconsidered Causes for Complications in Aesthetic Breast Augmentation	2022	ML: CART model: Machine learning with classification and regression tree analysis	AI identified patient and procedural risk factors to predict complications and improve safety	AI identified overlooked predictors (breast size, height, age) with high accuracy for complication risk across procedures
https://doi.org/10.1007/s00266-023-03432-w	Markovic et al.	Assessment of Three Breast Volume Measurement Techniques: Single Marking, MRI and Crisalix 3D Software®	2023	Computer vision: Crisalix®: Cloud-based 3D simulation software	AI was used to calculate breast volume pre operatively by generating 3D models from 2D images and sensor-based body mapping	Crisalix 3D AI software offers a practical, low-cost approach for breast volume estimation with limited accuracy in preoperative planning
https://doi.org/10.1007/s00266-024-04142-7	Huyghebaert et al.	Implementation of a Machine Learning Approach Evaluating Risk Factors for Complications after Single-Stage Augmentation Mastopexy	2024	ML: CART, RF and XGBoost: Ensemble machine learning combining CART, Random Forest, and XGBoost algorithms	Complication risks and factors driving implant exchange decisions	AI models accurately predicted complications and implant exchange decisions, with overall low complication rates and favorable outcomes

*DOI* Digital Object Identifier, *AI* artificial intelligence, *ANN* artificial neural network, *SVM* support vector machine, *BMI* body mass index, *CART* classification and regression tree, *RF* random forest, *XGBoost* extreme gradient boosting

## Discussion

AI, AR, and robotics are rapidly transforming healthcare by enhancing diagnostic precision, optimizing surgical planning, improving procedural accuracy, and enabling personalized treatment strategies [5, 16, 65]. In cosmetic medicine and surgery, these technologies are evolving from experimental tools to clinically relevant adjuncts, supporting both surgical and non-surgical interventions through objective analysis, risk stratification, and real-time intraoperative guidance [66–69]. Their integration holds the potential to improve safety, efficiency, and patient satisfaction, aligning cosmetic outcomes more closely with individualized goals. Given this growing importance, this systematic review aims to comprehensively evaluate current evidence-based data on their performance and clinical impact in cosmetic practice, with a particular focus on present-day clinical applicability.

It is important to acknowledge that the term AI is broad, and this technology is often associated with the two others reviewed here; this systematic review confirms that today’s clinicians can use these three tools before, during, and after cosmetic procedures.

During consultation and before any intervention, physicians can rely on ML algorithms to identify suitable candidates for cosmetic procedures [64] This results in a patient-centered cosmetic pathway, as demonstrated by Cazzaniga et al., who predicted better response rates in selected patients undergoing excimer laser treatment for reducing vitiligo spots as well as in studies predicting outcomes in aesthetic breast augmentation and rhinoplasty [39, 40, 44, 45, 61]. Such tools also enhance communication with patients regarding risks and expected benefits. With regards to cosmetic procedures, these tools importantly show a visual expectation that can be evaluated collaboratively between the patient and practitioner, akin to the use of transfer paper prior to tattooing. This likely helps mitigate ambiguity and manage expectations.

Through validated and peer-reviewed software, aesthetic physicians and surgeons can now conduct computer vision and ML-assisted clinical examinations, offering more precise and reproducible skin analyses, along with 3D modeling that helps both practitioner and patient target specific areas of concern [27, 29, 30, 34, 70, 71]. AR also supports communication and decision-making during consultation. For example, the Arbrea® Breast Software combines AR and ML to facilitate shared physician–patient decision-making regarding implant selection, leading to increased patient satisfaction [54]. Moreover, in aesthetic medicine, virtual reality enables training of physicians in filler injection techniques [60]. Together, AI and AR are emerging as genuine decision-support and communication tools in the clinic.

During the cosmetic procedure itself, real-time data analysis can guide the physician’s actions. For example, in cryolipolysis, devices equipped with USER ASSIST technology automatically adjust treatment parameters to modulate intensity [43]. Similarly, in orthognathic surgery, AR-assisted intraoperative navigation ensures closer adherence to preoperative planning. This is also where AR becomes highly relevant: in orthognathic aesthetic procedures, maxillofacial surgeons can now use head-mounted AR displays to project preoperative plans directly onto the patient [55–57]. By the same principle, aesthetic physicians can refine their techniques by following pre-injection mapping during botulinum toxin injections or by visualizing major facial arteries before filler placement [53, 58, 59].

At this stage, robotics also comes into play. In cosmetic procedures, robotic automation is currently applied mainly to repetitive, standardized tasks such as laser hair removal or hair restoration [51, 52]. In facial aesthetic surgery, robotics are being used to enhance safety and precision by adopting reconstructive techniques that minimize incision size and improve visualization with instrument-integrated cameras [72]. These early promising efforts to adopt and integrate AI, VR, and robotics in aesthetic medical practice give way to more precise, delicate, and safer surgeries with better outcomes. Thus far, cadaveric studies suggest that robotic assistance may soon be feasible in aesthetic surgery, particularly for deep-plane facelifts and complex rhinoplasties where precision and preservation of critical structures are paramount [46, 47].

During the post-procedure period, both physician and patient remain the ultimate judges of outcome. While patient satisfaction is paramount, ML tools can introduce a degree of objectivity. For instance, improvements in skin quality can be quantified using several commercially available software platforms [28, 32, 73, 74]. In aesthetic medicine and surgery, published applications of objective outcome assessment currently focus primarily on lip augmentation, rhinoplasty, orthognathic surgery and breast augmentation [31, 36–38, 75–78]. Finally, the ML-based CAARISMA® ARMM platform provides users with aesthetic and perceived-age indices, allowing for before-and-after comparisons offering a new objective post-procedure evaluation tool [2, 3].

When comparing these findings to the literature, current insights increasingly underscore the transformative potential of AI, AR, and robotics in cosmetic procedures [79]. AI applications have expanded from early image-based analysis and pattern recognition using classical computer vision to sophisticated, ML or DL multimodal systems capable of integrating demographic, clinical, and imaging data for personalized treatment planning and risk stratification [66, 80]. AR platforms have demonstrated value in improving procedural accuracy through real-time anatomical overlays and in patient engagement by visualizing likely outcomes before and during intervention [81]. All AR-related peer-reviewed studies focused on facial cosmetic surgery and medicine, highlighting a significant lack of evaluation of AR in body-contouring procedures.

The use of robotics in purely cosmetic procedures remains largely experimental. In facial cosmetic surgery, its development has struggled to gain a foothold as the few available studies, often of mediocre quality, do not provide sufficient evidence to determine their utility. Its aim, alongside AR, is to enhance precision, enable minimally invasive access, and improve safety through advanced visualization. This slower adoption compared with other specialties can be explained by the seemingly lower necessity for such technology in aesthetic surgery, as well as its high cost and the lack of specialized training [72, 82]. Advancements in aesthetic medicine exist as a company has developed a robotic arm capable of injecting hyaluronic acid; yet, no studies have investigated the performance of this device [83].

Comparing these three domains highlights clear differences in their stage of clinical translation. AI currently represents the most mature technology in aesthetic practice, with multiple validated applications for image analysis, surgical planning, and objective outcome assessment using systems such as VECTRA®-based volumetric analysis and ML-driven aesthetic scoring platforms. AR appears to be at an intermediate stage, with studies demonstrating promising accuracy for intraoperative navigation and anatomical visualization. However, these applications remain largely confined to research settings and have not yet achieved widespread clinical adoption as their adoption remains limited by challenges in real-time registration, hardware ergonomics, and workflow integration. In contrast, robotics remains largely experimental in aesthetic procedures, with current applications mostly limited to specialized tasks. These differences suggest that AI is currently driving clinical implementation, whereas AR and robotics are still progressing toward broader integration in aesthetic workflows.

Nevertheless, translating these benefits into routine practice will require overcoming technical, regulatory, and ethical barriers, while ensuring that patient safety and clinical efficacy remain paramount [84]. The accuracy and reproducibility of AI models are heavily dependent on dataset quality, which in aesthetic medicine is often limited by small sample sizes, lack of diversity because of geographical and demographic limitations and subjective outcome measures. Until now, these models are trained on geographically restricted datasets that may not reflect global variation in facial features, skin types, and aesthetic preferences. For example, Linming et al. evaluated skin imaging algorithms using datasets derived primarily from Chinese populations [29], while selfie-based datasets such as those analyzed by Georgievskaya et al. are often regionally concentrated and demographically imbalanced [35]. Similarly, AI facial attractiveness scoring systems such as Face++ rely largely on datasets derived from Asian populations [85]. In these studies, the authors suggest that aesthetic evaluations should therefore be interpreted within the same regional population; however, this approach risks perpetuating algorithmic bias rather than improving the generalizability of AI-based aesthetic assessments across diverse patient populations. [66, 69]. AR implementation can be hampered by workflow integration challenges, the need for precise registration accuracy in dynamic surgical fields, and the potential for over-reliance on visual augmentation at the expense of tactile skill development. Robotics introduces financial, logistical, and training burdens that may limit widespread adoption, particularly in smaller practices, and raises questions about liability in cases of device malfunction or algorithmic error [86–89]. In fact, the introduction of algorithm-based decision support, intraoperative guidance, or automated procedures inevitably raises questions about accountability in case of error or adverse outcomes.

As highlighted in recent literature, issues such as liability attribution between software developers, device manufacturers, and physicians remain unresolved [90]. From a legal and ethical standpoint, the use of AI and AR in aesthetic interventions demands heightened scrutiny regarding informed consent, data security, and the transparency of algorithmic decision-making [91]. The commercial nature of much of aesthetic medicine also raises concerns about the potential for over-promotion of technology-driven treatments without sufficient longitudinal outcome data [68, 92]. To address these ethical concerns, clear governance frameworks are needed for the clinical use of AI-assisted tools in aesthetic medicine. Informed consent should explicitly disclose the role of AI in consultation or treatment planning, including the nature of the algorithmic analysis, its limitations, and the potential for bias or prediction error. Patients should also be informed whether AI outputs function only as advisory tools or influence clinical recommendations. Notably, despite the growing number of AI platforms used in aesthetic consultations, none of them are formally approved as software as a medical device by regulatory agencies such as the U.S. Food and Drug Administration [93], with most systems marketed as consultation or visualization tools rather than clinical decision-support devices. Emerging regulatory frameworks, including the European Union Artificial Intelligence Act [94] and SaMD regulations, emphasize transparency, traceability, and human oversight. Within this model, developers remain responsible for algorithm validation and safety, while physicians retain final clinical decision-making authority. Nonetheless, when implemented within evidence-based frameworks, these technologies offer clear potential benefits: improved surgical precision, reduced complication rates, enhanced patient satisfaction, and the potential to democratize expertise through decision-support systems and remote guidance. In line with our findings, the literature supports the notion that integrating AI, AR, and robotics into cosmetic procedures can accelerate the shift toward personalized, data-driven care [67, 95, 96].

This review is subject to several limitations that reflect both the current state of the literature and inherent challenges in evaluating AI, AR, and robotics in cosmetic procedures. First, most included studies were based on small, heterogeneous patient cohorts, often lacking standardized outcome measures, which limits comparability and generalizability. Furthermore, training datasets for AI models were frequently restricted in size and demographic diversity, raising concerns regarding potential algorithmic bias, particularly when aesthetic norms and perceptions differ substantially across ethnicities, regions, and cultures. Regional variation in procedural preferences and outcome expectations further complicates the extrapolation of findings to a global context. The included study designs varied widely, ranging from early feasibility and technical validation studies to prospective cohort studies and randomized controlled trials. This heterogeneity in methodological approaches further limits the direct comparability and broader applicability of reported outcomes. Accordingly, methodological rigor was generally low to moderate with a predominance of retrospective designs and limited longitudinal follow-up, hindering the assessment of sustained clinical benefit or safety. Additionally, due to substantial study heterogeneity and the frequent absence of clearly reported numerical outcome data, quantitative synthesis was not feasible, and the GRADE approach was therefore applied to assess the certainty of evidence across included studies. The elective nature of cosmetic interventions amplifies ethical considerations around patient consent, data privacy, and the transparency of algorithmic decision-making, particularly in commercial settings where marketing pressures may influence technology adoption. The above factors limit how our findings can be applied in clinical practice and the conclusions that can be drawn from this evidence. As such, the reported results should be interpreted with caution and considered exploratory rather than definitive.

## Conclusion

AI, AR, and robotics can redefine aesthetic practice. Evolving from classical computer vision to DL, AI now supports advanced image analysis, risk prediction, and outcome simulation; AR improves surgical accuracy, procedural safety, and patient engagement; and robotics has the potential to deliver minimally invasive approaches with sub-millimeter precision and faster recovery although its implementation in aesthetic practice is not well documented. Their promise, however, is balanced by challenges in evidence quality, dataset diversity, workflow integration, costs, training, and ethical oversight. Given the scarcity and limited rigor of current studies, findings should be interpreted cautiously, and clinical benefits remain uncertain. Wider adoption requires robust trials, standardized protocols, transparent algorithms, and strong regulation to ensure equitable, evidence-based innovation in cosmetic procedures. Applied responsibly, these tools aim to augment, without replacing, clinical expertise.

## Supplementary Information

Below is the link to the electronic supplementary material.

Supplementary file1 (DOCX 75 KB)
