# Supplementary material for: Technological Integration in Aesthetic Practice: A Systematic Review of Artificial Intelligence, Augmented Reality and Robotics in Cosmetic Procedures
Source: Aesthetic Plast Surg. 2026 May 15;50(13):5643–64. doi: 10.1007/s00266-026-05898-w (PMC13433551; doi:10.1007/s00266-026-05898-w)
Supplement: Supplementary file 1 [file 266_2026_5898_MOESM1_ESM.docx]

**Supplemental Digital Content**

**Supplemental Digital Content 1.** Full search strategy for all databases included.

| **Database** | **Search String** | **Results** |
| --- | --- | --- |
| PubMed/MEDLINE | ("artificial intelligence"[MeSH Terms] OR "machine learning"[MeSH Terms] OR "deep learning" OR "AI" OR "robotics"[MeSH Terms] OR "robotic surgical procedures"[MeSH Terms] OR "robot-assisted" OR "augmented reality" OR "AR") AND ("cosmetic techniques"[MeSH Terms] OR "aesthetic procedures" OR "cosmetic procedures" OR "plastic surgery" OR "esthetic surgery" OR "reconstructive surgical procedures"[MeSH Terms]) | 3,911 |
| Google Scholar (first 10 pages) | ("artificial intelligence" OR "machine learning" OR "deep learning" OR "AI" OR "robotics" OR "robot-assisted" OR "augmented reality" OR "AR") AND ("cosmetic procedures" OR "aesthetic procedures" OR "plastic surgery" OR "esthetic surgery" OR "cosmetic surgery") | 250 |
| Web of Science | ("artificial intelligence" OR "machine learning" OR "deep learning" OR AI OR robotics OR "robot-assisted" OR "augmented reality" OR AR) AND ("cosmetic procedure*" OR "aesthetic procedure*" OR "esthetic procedure*" OR "plastic surgery" OR "cosmetic surgery" OR "reconstructive surgery") | 1,521 |
| Embase | ('artificial intelligence'/exp OR 'machine learning'/exp OR 'deep learning' OR 'ai' OR 'robotics'/exp OR 'robot-assisted' OR 'augmented reality'/exp OR 'ar') AND ('cosmetic surgery'/exp OR 'aesthetic surgery' OR 'esthetic surgery' OR 'plastic surgery'/exp OR 'cosmetic procedure*' OR 'aesthetic procedure*') | 6,634 |

**Supplementary Digital Content 2.** Quality assessment using the Newcastle-Ottawa Scale scoring system. Studies are listed in alphabetical order of first author.

| **DOI** | **First author** | **Title** | **Study type** | **Selection** | **Comparability** | **Exposure** | **NOS-score** |
| --- | --- | --- | --- | --- | --- | --- | --- |
| DOI: 10.1097/PRS.0000000000003433 | Adjadj et al. | Assessment of the Efficacy of Cryolipolysis on Saddlebags: A Prospective Study of 53 Patients | Prospective study | 2 | 0 | 2 | 4 |
| DOI: 10.1097/GOX.0000000000003698 | Bukret et al. | A Novel Artificial Intelligence–assisted Risk Assessment Model for Preventing Complications in Esthetic Surgery | Retrospective analysis | 2 | 0 | 2 | 4 |
| DOI: 10.1097/SAP.0000000000002637 | Chen et al. | A Comparative Study of Three-Dimensional Simulation in Nonsurgical Rhinoplasty With Hyaluronic Acid Fillers | Retrospective comparative study | 2 | 1 | 2 | 5 |
| DOI: 10.1055/s-0041-1729911 | Chinski et al. | An Artificial Intelligence Tool for Image Simulation in Rhinoplasty | Cross-sectional survey study | 1 | 0 | 2 | 3 |
| DOI: 10.1093/asj/sjae040 | Demir et al. | Optimizing Implant Width Selection in Breast Augmentation: Insights From On-Patient Landmark Positioning in 3-Dimensional Breast Simulation | Retrospective observational study | 2 | 0 | 2 | 4 |
| DOI: 10.1097/PRS.0000000000009671 | Georgievskaya et al. | Artificial Intelligence Confirming Treatment Success: The Role of Gender- and Age-Specific Scales in Performance Evaluation | Observational study using retrospective analysis of anonymized image data | 2 | 0 | 2 | 4 |
| DOI: 10.1111/jocd.14106 | Holcomb et al. | Helium plasma dermal resurfacing: VISIA CR assessment of facial spots, pores, and wrinkles—Preliminary findings | Single-arm prospective, multicenter clinical study (post hoc subanalysis) | 3 | 0 | 2 | 5 |
| DOI: 10.1007/s00266-024-04142-7 | Huyghebaert et al. | Implementation of a Machine Learning Approach Evaluating Risk Factors for Complications after Single-Stage Augmentation Mastopexy | Retrospective study | 2 | 0 | 2 | 4 |
| DOI: 10.1007/s00266-025-04891-z | Kang et al. | AI-Driven Assessment of Lip Volume Improvement Using Hyaluronic Acid Fillers: A Comprehensive Analysis | Prospective observational study | 3 | 0 | 2 | 5 |
| DOI: 10.2147/CCID.S135441 | Kerscher et al. | Effectiveness evaluation of two volumizing hyaluronic acid dermal fillers in a controlled, randomized, double-blind, split-face clinical study | Controlled, randomized, double-blind, split-face clinical study | 3 | 2 | 3 | 8 |
| DOI: 10.1038/s41598-023-49006-3 | Knoedler et al. | Diagnosing lagophthalmos using artificial intelligence | Prospective observational study | 3 | 0 | 2 | 5 |
| DOI: 10.1016/j.jormas.2024.102211 | Knoedler et al. | Objective and automated facial palsy grading and outcome assessment after facial palsy reanimation surgery – A prospective observational study | Prospective observational study | 3 | 0 | 2 | 5 |
| DOI: 10.1016/j.jormas.2025.102277 | Knoedler et al. | Objectifying aesthetic outcomes following face transplantation – the AI research metrics model (CAARISMA® ARMM) | Prospective observational study | 3 | 0 | 2 | 5 |
| DOI: 10.3390/jcm11123464 | La Padula et al. | Assessment of Patient Satisfaction Using a New Augmented Reality Simulation Software for Breast Augmentation: A Prospective Study | Prospective cohort study | 2 | 0 | 2 | 4 |
| DOI: 10.21037/gs-24-148 | Lee et al. | Prediction model for intraoperative implant volume using the 3D surface imaging system (VECTRA XT 3D) in direct-to-implant breast reconstructions | Retrospective observational study | 2 | 0 | 2 | 4 |
| DOI: 10.1089/pho.2015.3948 | Lim et al. | Comparison of Efficacy Between Novel Robot-Assisted Laser Hair Removal and Physician-Directed Hair Removal | Clinical trial | 4 | 1 | 3 | 8 |
| DOI: 10.1038/s41598-021-85889-w | Lin et al. | Preliminary clinical experience of robot-assisted surgery in treatment with genioplasty | Preliminary clinical study | 2 | 0 | 2 | 4 |
| DOI: 10.1007/s00266-023-03432-w | Markovic et al. | Assessment of Three Breast Volume Measurement Techniques: Single Marking, MRI and Crisalix 3D Software® | Prospective cohort study | 2 | 1 | 2 | 5 |
| DOI: 10.1007/s00266-022-02997-2 | Montemurro et al. | A Machine Learning Approach to Identify Previously Unconsidered Causes for Complications in Aesthetic Breast Augmentation | Retrospective study | 2 | 0 | 2 | 4 |
| DOI: 10.1111/srt.12464 | Petit et al. | Validation of 3D skin imaging for objective repeatable quantification of severity of atrophic acne scarring | Prospective, non-interventional, single-center validation study | 2 | 0 | 2 | 4 |
| DOI: 10.1186/s13005-024-00406-4 | Ritschl et al. | Comparison of three-dimensional imaging of the nose using three different 3D-photography systems: an observational study | Observational study | 2 | 0 | 2 | 4 |
| DOI: 10.1093/asj/sjab195 | Sforza et al. | Hybrid Implant and Grafted Fat Breast Augmentation: Designing the Pathway to a Future With Breasts Free of Silicone Breast Implants | Prospective cohort study | 2 | 0 | 2 | 4 |
| PMID: 29601620 | Taub et al. | Multi-Center, Double-Blind, Vehicle-Controlled Clinical Trial of an Alpha and Beta Defensin-Containing Anti-Aging Skin Care Regimen | Multi-center, double-blind, placebo-controlled clinical trial | 3 | 2 | 3 | 8 |
| DOI: 10.1097/PRS.0000000000008670 | van Mulken et al. | One-Year Outcomes of the First Human Trial on Robot-Assisted Lymphaticovenous Anastomosis for Breast Cancer–Related Lymphedema | Prospective randomized pilot study | 3 | 1 | 3 | 7 |
| DOI: 10.1007/s00266-017-0830-2 | Vorstenbosch et al. | Correlation of Prediction and Actual Outcome of Three-Dimensional Simulation in Breast Augmentation Using a Cloud-Based Program | Retrospective observational study | 2 | 1 | 2 | 5 |
| DOI: 10.1111/jocd.16554 | Zhu et al. | A Comparative Study on the Application of Robotic Hair Restoration Technology Versus Traditional Follicular Unit Excision in Male Androgenetic Alopecia | Single-center, prospective, randomized, single-blinded, controlled, split-scalp pilot study | 4 | 1 | 3 | 8 |
| DOI: 10.1016/j.bjoms.2013.06.014 | Zinser et al. | Computer-assisted orthognathic surgery: waferless maxillary positioning, versatility, and accuracy of an image-guided visualisation display | Observational study | 2 | 0 | 2 | 4 |

**Supplementary Digital Content 3.** Quality assessment using the Level of Evidence scoring system.

| **DOI** | **First author** | **Title** | **Study type** | **Level of Evidence** |
| --- | --- | --- | --- | --- |
| DOI: 10.1097/PRS.0000000000003433 | Adjadj et al. | Assessment of the Efficacy of Cryolipolysis on Saddlebags: A Prospective Study of 53 Patients | Prospective study | 3 |
| DOI: 10.14569/IJACSA.2019.0100941 | Alrabiah et al. | Computer-based Approach to Detect Wrinkles and Suggest Facial Fillers | Experimental study (methodological framework development and evaluation) | 5 |
| DOI: 10.1016/j.jcms.2014.09.001 | Badiali et al. | Augmented reality as an aid in maxillofacial surgery: Validation of a wearable system allowing maxillary repositioning | Feasibility and validation study (in vitro) | 5 |
| DOI: 10.1080/2000656X.2021.2024553 | Bai et al. | Clinical assessment of breast symmetry and aesthetic outcome: can 3D imaging be the gold standard? | Methodological study | 5 |
| DOI: 10.1016/j.patcog.2014.08.003 | Batool et al. | Fast detection of facial wrinkles based on Gabor features using image morphology and geometric constraints | Algorithm development and validation study (image processing/computer vision research) | 5 |
| DOI: 10.1097/GOX.0000000000003698 | Bukret et al. | A Novel Artificial Intelligence–assisted Risk Assessment Model for Preventing Complications in Esthetic Surgery | Retrospective analysis | 4 |
| DOI: 10.1159/000225934 | Cazzaniga et al. | Prediction of Clinical Response to Excimer Laser Treatment in Vitiligo by Using Neural Network Models | Predictive modeling study based on a previously conducted randomized controlled trial | 5 |
| DOI: 10.1093/asjof/ojab052 | Chartier et al. | BreastGAN: Artificial Intelligence-Enabled Breast Augmentation Simulation | Retrospective image analysis | 4 |
| DOI: 10.1097/SAP.0000000000002637 | Chen et al. | A Comparative Study of Three-Dimensional Simulation in Nonsurgical Rhinoplasty With Hyaluronic Acid Fillers | Retrospective comparative study | 3 |
| DOI: 10.1055/s-0041-1729911 | Chinski et al. | An Artificial Intelligence Tool for Image Simulation in Rhinoplasty | Cross-sectional survey study | 3 |
| DOI: 10.1093/asj/sjae040 | Demir et al. | Optimizing Implant Width Selection in Breast Augmentation: Insights From On-Patient Landmark Positioning in 3-Dimensional Breast Simulation | Retrospective observational study | 3 |
| DOI: 10.1093/asj/sjz259 | Dorfman et al. | Making the Subjective Objective: Machine Learning and Rhinoplasty | Retrospective chart review with AI-based image analysis | 4 |
| DOI: 10.1016/j.jid.2020.08.027 | Dulmage et al. | A Point-of-Care, Real-Time Artificial Intelligence System to Support Clinician Diagnosis of a Wide Range of Skin Diseases | Diagnostic accuracy study and AI system validation | 5 |
| DOI: 10.3390/jimaging6040017 | Elbashir et al. | Evaluation of Automatic Facial Wrinkle Detection Algorithms | Comparative study / Diagnostic evaluation | 4 |
| DOI: 10.1111/srt.13206 | Fujino et al. | Real-time wrinkle evaluation method using Visual Illusion-based image feature enhancement System | Original research article / method development and validation study | 5 |
| DOI: 10.1097/PRS.0000000000009671 | Georgievskaya et al. | Artificial Intelligence Confirming Treatment Success: The Role of Gender- and Age-Specific Scales in Performance Evaluation | Observational study using retrospective analysis of anonymized image data | 3 |
| DOI: 10.1111/jocd.14106 | Holcomb et al. | Helium plasma dermal resurfacing: VISIA CR assessment of facial spots, pores, and wrinkles—Preliminary findings | Single-arm prospective, multicenter clinical study (post hoc subanalysis) | 3 |
| DOI: 10.1038/s41598-024-63274-7 | Huang et al. | A comparative study of an advanced skin imaging system in diagnosing facial pigmentary and inflammatory conditions | Comparative study / Diagnostic evaluation | 4 |
| DOI: 10.1007/s00266-024-04142-7 | Huyghebaert et al. | Implementation of a Machine Learning Approach Evaluating Risk Factors for Complications after Single-Stage Augmentation Mastopexy | Retrospective study | 4 |
| DOI: 10.1007/s00266-025-04891-z | Kang et al. | AI-Driven Assessment of Lip Volume Improvement Using Hyaluronic Acid Fillers: A Comprehensive Analysis | Prospective observational study | 3 |
| DOI: 10.2147/CCID.S135441 | Kerscher et al. | Effectiveness evaluation of two volumizing hyaluronic acid dermal fillers in a controlled, randomized, double-blind, split-face clinical study | Controlled, randomized, double-blind, split-face clinical study | 2 |
| DOI: 10.1002/cpe.5526 | Kim et al. | Augmented reality for botulinum toxin injection | Technical implementation and evaluation study | 5 |
| DOI: 10.1038/s41598-023-49006-3 | Knoedler et al. | Diagnosing lagophthalmos using artificial intelligence | Prospective observational study | 3 |
| DOI: 10.1016/j.jormas.2024.102211 | Knoedler et al. | Objective and automated facial palsy grading and outcome assessment after facial palsy reanimation surgery – A prospective observational study | Prospective observational study | 3 |
| DOI: 10.1016/j.jormas.2025.102277 | Knoedler et al. | Objectifying aesthetic outcomes following face transplantation – the AI research metrics model (CAARISMA® ARMM) | Prospective observational study | 3 |
| DOI: 10.3390/jcm11123464 | La Padula et al. | Assessment of Patient Satisfaction Using a New Augmented Reality Simulation Software for Breast Augmentation: A Prospective Study | Prospective cohort study | 3 |
| DOI: 10.21037/gs-24-148 | Lee et al. | Prediction model for intraoperative implant volume using the 3D surface imaging system (VECTRA XT 3D) in direct-to-implant breast reconstructions | Retrospective observational study | 3 |
| DOI: 10.1111/jocd.16218 | Lee et al. | Deep learning-based skin care product recommendation: A focus on cosmetic ingredient analysis and facial skin conditions | Original Article | 5 |
| DOI: 10.1007/s00266-025-04876-y | Li et al. | An Artificial Intelligence Tool Used for Patient Selection in Cosmetic Surgery | Prospective validation study | 4 |
| DOI: 10.1089/pho.2014.3774 | Lim et al. | A Study on the Development of a Robot-Assisted Automatic Laser Hair Removal System | Prototype development and technical validation study | 5 |
| DOI: 10.1089/pho.2015.3948 | Lim et al. | Comparison of Efficacy Between Novel Robot-Assisted Laser Hair Removal and Physician-Directed Hair Removal | Clinical trial | 2 |
| DOI: 10.1016/j.jcms.2015.10.024 | Lin et al. | Mandibular angle split osteotomy based on a novel augmented reality navigation using specialized robot-assisted arms – A feasibility study | Feasibility study | 5 |
| DOI: 10.1038/s41598-021-85889-w | Lin et al. | Preliminary clinical experience of robot-assisted surgery in treatment with genioplasty | Preliminary clinical study | 4 |
| DOI: 10.1111/srt.12381 | Linming et al. | Comparison of two skin imaging analysis instruments: The VISIA® from Canfield vs the ANTERA 3D®CS from Miravex | Comparative study / Diagnostic evaluation | 4 |
| DOI: 10.1007/s00266-023-03432-w | Markovic et al. | Assessment of Three Breast Volume Measurement Techniques: Single Marking, MRI and Crisalix 3D Software® | Prospective cohort study | 3 |
| DOI: 10.1093/asjof/ojab018 | Mespreuve et al. | The Usefulness of Magnetic Resonance Angiography to Analyze the Variable Arterial Facial Anatomy in an Effort to Reduce Filler-Associated Blindness: Anatomical Study and Visualization Through an Augmented Reality Application | Anatomical study (prospective volunteer-based imaging study) | 4 |
| DOI: 10.1245/s10434-013-3470-z | Mohamed et al. | Retroauricular Robotic Thyroidectomy with Concomitant Neck-Lift Surgery | Case report | 4 |
| DOI: 10.1007/s00266-022-02997-2 | Montemurro et al. | A Machine Learning Approach to Identify Previously Unconsidered Causes for Complications in Aesthetic Breast Augmentation | Retrospective study | 4 |
| DOI: 10.1007/s00266-020-01872-2 | Oh et al. | Development and Usability of a Virtual Reality-Based Filler Injection Training System | Prospective usability study | 4 |
| DOI: 10.1089/pho.2015.4018 | Park et al. | Improvement in Laser-Irradiation Efficiency of Robot-Assisted Laser Hair Removal Through Pose Measurement of Skin Surface | Technical improvement study (experimental system evaluation) | 5 |
| DOI: 10.1111/srt.12464 | Petit et al. | Validation of 3D skin imaging for objective repeatable quantification of severity of atrophic acne scarring | Prospective, non-interventional, single-center validation study | 3 |
| DOI: 10.1186/s13005-024-00406-4 | Ritschl et al. | Comparison of three-dimensional imaging of the nose using three different 3D-photography systems: an observational study | Observational study | 3 |
| DOI: 10.1016/j.jormas.2019.03.012 | Savoldelli et al. | Accuracy, repeatability and reproducibility of a handheld three-dimensional facial imaging device: The Vectra H1 | Original article; Experimental validation study | 4 |
| DOI: 10.1093/asj/sjab195 | Sforza et al. | Hybrid Implant and Grafted Fat Breast Augmentation: Designing the Pathway to a Future With Breasts Free of Silicone Breast Implants | Prospective cohort study | 3 |
| DOI: 10.1097/SCS.0000000000009272 | Smith et al. | Robot-Assisted Nasal Reconstruction: A Cadaveric Study | Cadaveric study | 5 |
| DOI: 10.1007/s11701-013-0431-2 | Taghizadeh et al. | Evaluation of robotic-assisted platysmaplasty procedures in a cadaveric model using the da Vinci Surgical System | Cadaveric feasibility study | 5 |
| PMID: 29601620 | Taub et al. | Multi-Center, Double-Blind, Vehicle-Controlled Clinical Trial of an Alpha and Beta Defensin-Containing Anti-Aging Skin Care Regimen | Multi-center, double-blind, placebo-controlled clinical trial | 1b |
| DOI: 10.1097/PRS.0000000000008670 | van Mulken et al. | One-Year Outcomes of the First Human Trial on Robot-Assisted Lymphaticovenous Anastomosis for Breast Cancer–Related Lymphedema | Prospective randomized pilot study | 3 |
| DOI: 10.1007/s00266-017-0830-2 | Vorstenbosch et al. | Correlation of Prediction and Actual Outcome of Three-Dimensional Simulation in Breast Augmentation Using a Cloud-Based Program | Retrospective observational study | 3 |
| DOI: 10.1093/asjof/ojac012 | Waked et al. | Visualizing the Individual Arterial Anatomy of the Face Through Augmented Reality—A Useful and Accurate Tool During Dermal Filler Injections | Prospective clinical imaging accuracy study | 4 |
| DOI: 10.1002/rcs.1754 | Wang et al. | Video see-through augmented reality for oral and maxillofacial surgery | Original article (experimental system development and validation) | 5 |
| DOI: 10.1016/j.bjps.2025.02.014 | Wang et al. | Can AI-assisted objective facial attractiveness scoring systems replace manual aesthetic evaluations? A comparative analysis of human and machine ratings | Comparative analysis / validation study | 4 |
| DOI: 10.2147/CCID.S380388 | Zawodny et al. | VISIA Skin Analysis System as a Tool to Evaluate the Reduction of Pigmented Skin and Vascular Lesions Using the 532 Nm Laser | Comparative study / Diagnostic evaluation | 4 |
| DOI: 10.1111/jocd.16554 | Zhu et al. | A Comparative Study on the Application of Robotic Hair Restoration Technology Versus Traditional Follicular Unit Excision in Male Androgenetic Alopecia | Single-center, prospective, randomized, single-blinded, controlled, split-scalp pilot study | 3 |
| DOI: 10.1016/j.bjoms.2013.06.014 | Zinser et al. | Computer-assisted orthognathic surgery: waferless maxillary positioning, versatility, and accuracy of an image-guided visualization display | Observational study | 3 |

**Supplementary Digital Content 4** GRADE-rating for each study due to heterogeneity in study outcomes and lack of numerical data

| **Study Name** | **Study type** | **Risk of Bias** | **Inconsistency** | **Indirectness** | **Imprecision** | **Publication Bias** | **Other Considerations** | **Overall Certainty** |
| --- | --- | --- | --- | --- | --- | --- | --- | --- |
| **Artificial intelligence (AI)** |  |  |  |  |  |  |  |  |
| Batool 2015 | Algorithm development and validation study | Moderate | Not serious | Not serious | Serious | Suspected | Limited generalizability | ⊕⊕◯◯ Low |
| Kerscher 2017 | Controlled, randomized, double-blind, split-face clinical study | Moderate | Not serious | Not serious | Serious | Suspected | Limited generalizability | ⊕⊕◯◯ Low |
| Linming 2018 | Comparative study / diagnostic evaluation | High | Not serious | Serious | Serious | Suspected | Limited generalizability | ⊕◯◯◯ Very low |
| Petit 2018 | Prospective, non-interventional, single-center validation study | Moderate | Not serious | Not serious | Serious | Suspected | Limited generalizability | ⊕⊕◯◯ Low |
| Taub 2018 | Multi-Center, double-blind, vehicle-controlled clinical trial | Moderate | Not serious | Not serious | Serious | Suspected | Limited generalizability | ⊕⊕◯◯ Low |
| Savoldelli 2019 | Experimental validation study | Moderate | Not serious | Not serious | Serious | Suspected | Limited generalizability | ⊕⊕◯◯ Low |
| Alrabiah 2019 | Experimental validation study | Moderate | Not serious | Not serious | Serious | Suspected | Limited generalizability | ⊕⊕◯◯ Low |
| Elbashir 2020 | Comparative study / diagnostic evaluation | Moderate | Not serious | Not serious | Serious | Suspected | Limited generalizability | ⊕⊕◯◯ Low |
| Dorfman 2020 | Retrospective chart review with AI-based image analysis | Moderate | Not serious | Not serious | Serious | Suspected | Limited generalizability | ⊕⊕◯◯ Low |
| Chen 2021 | Retrospective comparative study | Moderate | Not serious | Not serious | Serious | Suspected | Limited generalizability | ⊕⊕◯◯ Low |
| Sforza 2021 | Prospective cohort study | High | Not serious | Not serious | Serious | Suspected | Limited generalizability | ⊕◯◯◯ Very low |
| Chinski 2021 | AI-based image analysis | Moderate | Not serious | Not serious | Serious | Suspected | Limited generalizability | ⊕⊕◯◯ Low |
| Holcomb 2021 | Single-arm prospective, multicenter clinical study | High | Not serious | Not serious | Serious | Suspected | Limited generalizability | ⊕◯◯◯ Very low |
| Dulmage 2021 | Diagnostic accuracy study and AI system validation | High | Not serious | Serious | Serious | Suspected | Limited generalizability | ⊕◯◯◯ Very low |
| Zawodny 2022 | Comparative study / diagnostic evaluation | High | Not serious | Not serious | Serious | Suspected | Limited generalizability | ⊕◯◯◯ Very low |
| Chartier 2022 BreastGAN | Retrospective AI-image analysis | Moderate | Not serious | Not serious | Serious | Suspected | Limited generalizability | ⊕⊕◯◯ Low |
| Georgievskaya 2022 | Observational study using retrospective analysis of anonymized image data | Moderate | Not serious | Not serious | Serious | Suspected | Limited generalizability | ⊕⊕◯◯ Low |
| Bai 2023 | Method development and validation study | High | Not serious | Serious | Serious | Suspected | Limited generalizability | ⊕◯◯◯ Very low |
| Knoedler 2023 lagophthalmos | Prospective observational study | High | Not serious | Serious | Serious | Suspected | Limited generalizability | ⊕◯◯◯ Very low |
| Fujino 2023 | Method development and validation study | Moderate | Not serious | Not serious | Serious | Suspected | Limited generalizability | ⊕⊕◯◯ Low |
| Ritschl 2024 | Observational study | High | Not serious | Serious | Serious | Suspected | Limited generalizability | ⊕◯◯◯ Very low |
| Lee 2024 implant | Retrospective observational study | High | Not serious | Serious | Serious | Suspected | Limited generalizability | ⊕◯◯◯ Very low |
| Demir 2024 | Retrospective observational study | High | Not serious | Serious | Serious | Suspected | Limited generalizability | ⊕◯◯◯ Very low |
| Huang 2024 | Comparative study / diagnostic evaluation | High | Not serious | Serious | Serious | Suspected | Limited generalizability | ⊕◯◯◯ Very low |
| Lee 2024 LUMINI | Method development and validation study | Moderate | Not serious | Not serious | Serious | Suspected | Limited generalizability | ⊕⊕◯◯ Low |
| Li 2025 | Prospective validation study | High | Not serious | Serious | Serious | Suspected | Limited generalizability | ⊕◯◯◯ Very low |
| Wang 2025 | Comparative analysis / validation study | Moderate | Not serious | Not serious | Serious | Suspected | Limited generalizability | ⊕⊕◯◯ Low |
| Kang 2025 | Prospective observational study | High | Not serious | Not serious | Serious | Suspected | Limited generalizability | ⊕◯◯◯ Very low |
| Knoedler 2025 palsy | Prospective observational study | High | Not serious | Serious | Serious | Suspected | Limited generalizability | ⊕◯◯◯ Very low |
| Knoedler 2025 transplant | Prospective observational study | High | Not serious | Serious | Serious | Suspected | Limited generalizability | ⊕◯◯◯ Very low |
| **Augmented reality (AR)** |  |  |  |  |  |  |  |  |
| Zinser 2013 | Observational study | High | Not serious | Serious | Serious | Suspected | Limited generalizability | ⊕◯◯◯ Very low |
| Badiali 2014 | Feasibility and validation study | Moderate | Not serious | Not serious | Serious | Suspected | Limited generalizability | ⊕⊕◯◯ Low |
| Wang 2016 | Experimental system development and validation | High | Not serious | Serious | Serious | Suspected | Limited generalizability | ⊕◯◯◯ Very low |
| Lin 2016 | Feasibility study | Moderate | Not serious | Not serious | Serious | Suspected | Limited generalizability | ⊕⊕◯◯ Low |
| Kim 2020 | Technical implementation and evaluation study | Moderate | Not serious | Not serious | Serious | Suspected | Limited generalizability | ⊕⊕◯◯ Low |
| Oh 2020 | Prospective feasibility study | Moderate | Not serious | Not serious | Serious | Suspected | Limited generalizability | ⊕⊕◯◯ Low |
| Mespreuve 2021 | Anatomical study (prospective volunteer-based imaging study) | Moderate | Not serious | Not serious | Serious | Suspected | Limited generalizability | ⊕⊕◯◯ Low |
| La Padula 2022 | Prospective cohort study | Moderate | Not serious | Not serious | Serious | Suspected | Limited generalizability | ⊕⊕◯◯ Low |
| Waked 2022 | Prospective clinical imaging accuracy study | Moderate | Not serious | Not serious | Serious | Suspected | Limited generalizability | ⊕⊕◯◯ Low |
| **Robotics** |  |  |  |  |  |  |  |  |
| Taghizadeh 2014 | Cadaveric feasibility study | High | Not serious | Serious | Serious | Suspected | Limited generalizability | ⊕◯◯◯ Very low |
| Lim 2014 | Prototype development and technical validation study | Moderate | Not serious | Serious | Serious | Suspected | Limited generalizability | ⊕⊕◯◯ Low |
| Mohamed 2015 | Case report | High | Not serious | Not serious | Serious | Suspected | Limited generalizability | ⊕◯◯◯ Very low |
| Lim 2015 | Clinical trial | Moderate | Not serious | Not serious | Serious | Suspected | Limited generalizability | ⊕⊕◯◯ Low |
| Park 2016 | Technical improvement study (experimental system evaluation) | Moderate | Not serious | Serious | Serious | Suspected | Limited generalizability | ⊕⊕◯◯ Low |
| Lin 2021 | Preliminary clinical study | High | Not serious | Serious | Serious | Suspected | Limited generalizability | ⊕◯◯◯ Very low |
| van Mulken 2022 | Prospective randomized pilot study | Moderate | Not serious | Not serious | Serious | Suspected | Limited generalizability | ⊕⊕◯◯ Low |
| Smith 2023 | Cadaveric study | High | Not serious | Serious | Serious | Suspected | Limited generalizability | ⊕◯◯◯ Very low |
| Zhu 2024 | Single-center, prospective, randomized, single-blinded, controlled, split-scalp pilot study | Moderate | Not serious | Not serious | Serious | Suspected | Limited generalizability | ⊕⊕◯◯ Low |

**Supplementary Digital Content 5.** Overview of study demographics and field of application of AI, AR, and robotics in cosmetic procedures.

| **DOI** | **First author** | **Title** | **Year of publication** | **Region of publication** | **Procedure type** | **Cosmetic Field** | **AI, AR or robotics** |
| --- | --- | --- | --- | --- | --- | --- | --- |
| DOI: 10.1097/PRS.0000000000003433 | Adjadj et al. | Assessment of the Efficacy of Cryolipolysis on Saddlebags: A Prospective Study of 53 Patients | 2017 | France | Cryolipolysis for saddlebag fat reduction | Aesthetic medicine | AI |
| DOI: 10.14569/IJACSA.2019.0100941 | Alrabiah et al. | Computer-based Approach to Detect Wrinkles and Suggest Facial Fillers | 2019 | Saudi Arabia | Wrinkle detection for filler planning (forehead) | Skin cosmetic assessment | AI |
| DOI: 10.1016/j.jcms.2014.09.001 | Badiali et al. | Augmented reality as an aid in maxillofacial surgery: Validation of a wearable system allowing maxillary repositioning | 2014 | Italy | LeFort I osteotomy with waferless maxillary repositioning | Facial aesthetic surgery | AR |
| DOI: 10.1080/2000656X.2021.2024553 | Bai et al. | Clinical assessment of breast symmetry and aesthetic outcome: can 3D imaging be the gold standard? | 2023 | Sweden | Risk-reducing mastectomy with immediate reconstruction, Vectra XT symmetry assessment | Non-facial aesthetic surgery | AI |
| DOI: 10.1016/j.patcog.2014.08.003 | Batool et al. | Fast detection of facial wrinkles based on Gabor features using image morphology and geometric constraints | 2015 | USA | Facial wrinkle detection from 2D images | Skin cosmetic assessment | AI |
| DOI: 10.1097/GOX.0000000000003698 | Bukret et al. | A Novel Artificial Intelligence–assisted Risk Assessment Model for Preventing Complications in Esthetic Surgery | 2021 | Argentina | Multiple aesthetic surgeries (breast, lipo, blepharoplasty, abdominoplasty, rhinoplasty) | Non-facial aesthetic surgery | AI |
| DOI: 10.1159/000225934 | Cazzaniga et al. | Prediction of Clinical Response to Excimer Laser Treatment in Vitiligo by Using Neural Network Models | 2009 | Italy | Excimer laser for vitiligo (face/neck pigmentation) | Energy-based devices | AI |
| DOI: 10.1093/asjof/ojab052 | Chartier et al. | BreastGAN: Artificial Intelligence-Enabled Breast Augmentation Simulation | 2022 | Canada | Bilateral breast augmentation | Non-facial aesthetic surgery | AI |
| DOI: 10.1097/SAP.0000000000002637 | Chen et al. | A Comparative Study of Three-Dimensional Simulation in Nonsurgical Rhinoplasty With Hyaluronic Acid Fillers | 2021 | China | Nonsurgical rhinoplasty with hyaluronic acid fillers and 3D simulation | Aesthetic medicine | AI |
| DOI: 10.1055/s-0041-1729911 | Chinski et al. | An Artificial Intelligence Tool for Image Simulation in Rhinoplasty | 2021 | Argentina | Rhinoplasty | Facial aesthetic surgery | AI |
| DOI: 10.1093/asj/sjae040 | Demir et al. | Optimizing Implant Width Selection in Breast Augmentation: Insights From On-Patient Landmark Positioning in 3-Dimensional Breast Simulation | 2024 | Turkey | Primary breast augmentation (implants) | Non-facial aesthetic surgery | AI |
| DOI: 10.1093/asj/sjz259 | Dorfman et al. | Making the Subjective Objective: Machine Learning and Rhinoplasty | 2020 | USA | Open rhinoplasty | Facial aesthetic surgery | AI |
| DOI: 10.1016/j.jid.2020.08.027 | Dulmage et al. | A Point-of-Care, Real-Time Artificial Intelligence System to Support Clinician Diagnosis of a Wide Range of Skin Diseases | 2021 | USA | Morphology classification of dermatological lesions | Skin cosmetic assessment | AI |
| DOI: 10.3390/jimaging6040017 | Elbashir et al. | Evaluation of Automatic Facial Wrinkle Detection Algorithms | 2020 | Sudan | Automatic wrinkle detection from facial images | Skin cosmetic assessment | AI |
| DOI: 10.1111/srt.13206 | Fujino et al. | Real-time wrinkle evaluation method using Visual Illusion-based image feature enhancement System | 2023 | Japan | Wrinkle analysis at eye corners | Skin cosmetic assessment | AI |
| DOI: 10.1097/PRS.0000000000009671 | Georgievskaya et al. | Artificial Intelligence Confirming Treatment Success: The Role of Gender- and Age-Specific Scales in Performance Evaluation | 2022 | Estonia | Facial skin analysis from selfies | Skin cosmetic assessment | AI |
| DOI: 10.1111/jocd.14106 | Holcomb et al. | Helium plasma dermal resurfacing: VISIA CR assessment of facial spots, pores, and wrinkles—Preliminary findings | 2021 | USA | Helium plasma dermal resurfacing (spots, pores, wrinkles) | Energy-based devices | AI |
| DOI: 10.1038/s41598-024-63274-7 | Huang et al. | A comparative study of an advanced skin imaging system in diagnosing facial pigmentary and inflammatory conditions | 2024 | Taiwan | Skin analysis of pigmentary and inflammatory conditions | Skin cosmetic assessment | AI |
| DOI: 10.1007/s00266-024-04142-7 | Huyghebaert et al. | Implementation of a Machine Learning Approach Evaluating Risk Factors for Complications after Single-Stage Augmentation Mastopexy | 2024 | Germany | Single-stage augmentation mastopexy (implant and lift) | Non-facial aesthetic surgery | AI |
| DOI: 10.1007/s00266-025-04891-z | Kang et al. | AI-Driven Assessment of Lip Volume Improvement Using Hyaluronic Acid Fillers: A Comprehensive Analysis | 2025 | China | Lip augmentation with hyaluronic acid filler | Aesthetic medicine | AI |
| DOI: 10.2147/CCID.S135441 | Kerscher et al. | Effectiveness evaluation of two volumizing hyaluronic acid dermal fillers in a controlled, randomized, double-blind, split-face clinical study | 2017 | Germany | Midfacial volumization with dermal fillers (cheeks) | Aesthetic medicine | AI |
| DOI: 10.1002/cpe.5526 | Kim et al. | Augmented reality for botulinum toxin injection | 2020 | South Korea | Botulinum toxin injection (facial muscles) | Aesthetic medicine | AR |
| DOI: 10.1016/j.jormas.2024.102211 | Knoedler et al. | Objective and automated facial palsy grading and outcome assessment after facial palsy reanimation surgery – A prospective observational study | 2025 | Germany | Microsurgical gracilis transfer for facial palsy reanimation | Facial aesthetic surgery | AI |
| DOI: 10.1016/j.jormas.2025.102277 | Knoedler et al. | Objectifying aesthetic outcomes following face transplantation – the AI research metrics model (CAARISMA® ARMM) | 2025 | Germany | Face transplantation (vascularized composite allotransplant) | Facial aesthetic surgery | AI |
| DOI: 10.1038/s41598-023-49006-3 | Knoedler et al. | Diagnosing lagophthalmos using artificial intelligence | 2023 | Germany | Diagnosis of lagophthalmos | Skin cosmetic assessment | AI |
| DOI: 10.3390/jcm11123464 | La Padula et al. | Assessment of Patient Satisfaction Using a New Augmented Reality Simulation Software for Breast Augmentation: A Prospective Study | 2022 | Italy | Primary breast augmentation (implants) | Non-facial aesthetic surgery | AR |
| DOI: 10.1111/jocd.16218 | Lee et al. | Deep learning-based skin care product recommendation: A focus on cosmetic ingredient analysis and facial skin conditions | 2024 | South Korea | AI-based skin analysis with LUMINI V2 + ingredient evaluation | Skin cosmetic assessment | AI |
| DOI: 10.21037/gs-24-148 | Lee et al. | Prediction model for intraoperative implant volume using the 3D surface imaging system (VECTRA XT 3D) in direct-to-implant breast reconstructions | 2024 | South Korea | Direct-to-implant breast reconstruction | Non-facial aesthetic surgery | AI |
| DOI: 10.1007/s00266-025-04876-y | Li et al. | An Artificial Intelligence Tool Used for Patient Selection in Cosmetic Surgery | 2025 | China | Facial cosmetic surgery (patient suitability assessment) | Facial aesthetic surgery | AI |
| DOI: 10.1089/pho.2014.3774 | Lim et al. | A Study on the Development of a Robot-Assisted Automatic Laser Hair Removal System | 2014 | South Korea | Laser hair removal | Energy-based devices | Robotics |
| DOI: 10.1089/pho.2015.3948 | Lim et al. | Comparison of Efficacy Between Novel Robot-Assisted Laser Hair Removal and Physician-Directed Hair Removal | 2015 | South Korea | Laser hair removal (upper thighs, 810 nm diode) | Energy-based devices | Robotics |
| DOI: 10.1016/j.jcms.2015.10.024 | Lin et al. | Mandibular angle split osteotomy based on a novel augmented reality navigation using specialized robot-assisted arms – A feasibility study | 2016 | China | Mandibular angle split osteotomy (MASO) | Facial aesthetic surgery | AR |
| DOI: 10.1038/s41598-021-85889-w | Lin et al. | Preliminary clinical experience of robot-assisted surgery in treatment with genioplasty | 2021 | China | Genioplasty with robotic osteotomy and drilling | Facial aesthetic surgery | Robotics |
| DOI: 10.1111/srt.12381 | Linming et al. | Comparison of two skin imaging analysis instruments: The VISIA® from Canfield vs the ANTERA 3D®CS from Miravex | 2018 | China | Facial skin imaging (wrinkles, pigmentation, texture, pores, vessels) | Skin cosmetic assessment | AI |
| DOI: 10.1007/s00266-023-03432-w | Markovic et al. | Assessment of Three Breast Volume Measurement Techniques: Single Marking, MRI and Crisalix 3D Software® | 2023 | Brazil | Breast volume measurement in patients undergoing mammoplasty (breast reduction surgery) | Non-facial aesthetic surgery | AI |
| DOI: 10.1093/asjof/ojab018 | Mespreuve et al. | The Usefulness of Magnetic Resonance Angiography to Analyze the Variable Arterial Facial Anatomy in an Effort to Reduce Filler-Associated Blindness: Anatomical Study and Visualization Through an Augmented Reality Application | 2021 | Belgium | Dermal filler planning with MRA vascular imaging | Aesthetic medicine | AR |
| DOI: 10.1245/s10434-013-3470-z | Mohamed et al. | Retroauricular Robotic Thyroidectomy with Concomitant Neck-Lift Surgery | 2015 | USA | Robotic hemithyroidectomy with neck lift | Facial aesthetic surgery | Robotics |
| DOI: 10.1007/s00266-022-02997-2 | Montemurro et al. | A Machine Learning Approach to Identify Previously Unconsidered Causes for Complications in Aesthetic Breast Augmentation | 2022 | Sweden | Primary breast augmentation (implants) | Non-facial aesthetic surgery | AI |
| DOI: 10.1007/s00266-020-01872-2 | Oh et al. | Development and Usability of a Virtual Reality-Based Filler Injection Training System | 2020 | South Korea | Dermal filler injection (face) | Aesthetic medicine | AI |
| DOI: 10.1089/pho.2015.4018 | Park et al. | Improvement in Laser-Irradiation Efficiency of Robot-Assisted Laser Hair Removal Through Pose Measurement of Skin Surface | 2016 | South Korea | Laser hair removal | Energy-based devices | Robotics |
| DOI: 10.1111/srt.12464 | Petit et al. | Validation of 3D skin imaging for objective repeatable quantification of severity of atrophic acne scarring | 2018 | France | 3D imaging of acne scarring with LifeViz® Micro | Skin cosmetic assessment | AI |
| DOI: 10.1186/s13005-024-00406-4 | Ritschl et al. | Comparison of three-dimensional imaging of the nose using three different 3D-photography systems: an observational study | 2024 | Germany | Non-surgical 3D nose imaging | Skin cosmetic assessment | AI |
| DOI: 10.1016/j.jormas.2019.03.012 | Savoldelli et al. | Accuracy, repeatability and reproducibility of a handheld three-dimensional facial imaging device: The Vectra H1 | 2019 | France | 3D stereophotogrammetry (Vectra H1) for facial morphology | Skin cosmetic assessment | AI |
| DOI: 10.1093/asj/sjab195 | Sforza et al. | Hybrid Implant and Grafted Fat Breast Augmentation: Designing the Pathway to a Future With Breasts Free of Silicone Breast Implants | 2021 | United Kingdom | Hybrid breast augmentation (implants and fat graft) | Non-facial aesthetic surgery | AI |
| DOI: 10.1097/SCS.0000000000009272 | Smith et al. | Robot-Assisted Nasal Reconstruction: A Cadaveric Study | 2023 | Canada | Nasal reconstruction using rib cartilage | Facial aesthetic surgery | Robotics |
| DOI: 10.1007/s11701-013-0431-2 | Taghizadeh et al. | Evaluation of robotic-assisted platysmaplasty procedures in a cadaveric model using the da Vinci Surgical System | 2014 | USA | Robotic-assisted platysmaplasty with facelift and liposuction | Facial aesthetic surgery | Robotics |
| PMID: 29601620 | Taub et al. | Multi-Center, Double-Blind, Vehicle-Controlled Clinical Trial of an Alpha and Beta Defensin-Containing Anti-Aging Skin Care Regimen | 2018 | USA | Topical skincare regimen (serum, cream, mask) | Skin cosmetic assessment | AI |
| DOI: 10.1097/PRS.0000000000008670 | van Mulken et al. | One-Year Outcomes of the First Human Trial on Robot-Assisted Lymphaticovenous Anastomosis for Breast Cancer–Related Lymphedema | 2022 | Netherlands | Supermicrosurgical lymphaticovenous for lymphedema | Non-facial aesthetic surgery | Robotics |
| DOI: 10.1007/s00266-017-0830-2 | Vorstenbosch et al. | Correlation of Prediction and Actual Outcome of Three-Dimensional Simulation in Breast Augmentation Using a Cloud-Based Program | 2017 | Canada | Breast augmentation using smooth, round implants | Non-facial aesthetic surgery | AI |
| DOI: 10.1093/asjof/ojac012 | Waked et al. | Visualizing the Individual Arterial Anatomy of the Face Through Augmented Reality—A Useful and Accurate Tool During Dermal Filler Injections | 2022 | Belgium | Dermal filler injection planning (facial anatomy visualization) | Aesthetic medicine | AR |
| DOI: 10.1002/rcs.1754 | Wang et al. | Video see-through augmented reality for oral and maxillofacial surgery | 2016 | China | Oral and maxillofacial surgery with navigation (teeth, maxilla, mandible) | Facial aesthetic surgery | AR |
| DOI: 10.1016/j.bjps.2025.02.014 | Wang et al. | Can AI-assisted objective facial attractiveness scoring systems replace manual aesthetic evaluations? A comparative analysis of human and machine ratings | 2025 | China | Facial attractiveness analysis from photos | Skin cosmetic assessment | AI |
| DOI: 10.2147/CCID.S380388 | Zawodny et al. | VISIA Skin Analysis System as a Tool to Evaluate the Reduction of Pigmented Skin and Vascular Lesions Using the 532 Nm Laser | 2022 | Poland | Laser therapy (532 nm) with VISIA skin analysis | Energy-based devices | AI |
| DOI: 10.1111/jocd.16554 | Zhu et al. | A Comparative Study on the Application of Robotic Hair Restoration Technology Versus Traditional Follicular Unit Excision in Male Androgenetic Alopecia | 2024 | China | Hair transplantation (robotic vs. manual follicular unit excision) | Facial aesthetic surgery | Robotics |
| DOI: 10.1016/j.bjoms.2013.06.014 | Zinser et al. | Computer-assisted orthognathic surgery: waferless maxillary positioning, versatility, and accuracy of an image-guided visualization display | 2013 | Germany | Orthognathic surgery (bimaxillary osteotomy, maxillary positioning) | Facial aesthetic surgery | AI |
| *Notes: 2D, two-dimensional; 3D, three-dimensional; AI, artificial intelligence; AR, augmented reality; DOI, Digital Object Identifier; MASO, mandibular angle split osteotomy; MRA, magnetic resonance angiography; Nm, nanometer; PMID, PubMed identifier; USA, United States of America; UK, United Kingdom* | | | | | | | |
